# Perinatal exposure to synergistic multiple stressors leads to cellular and behavioral deficits mimicking Schizophrenia-like pathology

**DOI:** 10.1242/bio.058870

**Published:** 2022-03-09

**Authors:** Tiyasha Sarkar, Nisha Patro, Ishan Kumar Patro

**Affiliations:** School of Studies in Neuroscience, Jiwaji University, Gwalior 474011, India

**Keywords:** Multi-hit, Protein malnourishment, Poly I:C, Lipopolysaccharide (LPS), Synaptic plasticity proteins, Schizophrenia

## Abstract

Protein malnourishment and immune stress are potent perinatal stressors, encountered by children born under poor socioeconomic conditions. Thus, it is necessary to investigate how such stressors synergistically contribute towards developing neurological disorders in affected individuals. Pups from Wistar females, maintained on normal (high-protein, HP:20%) and low-protein (LP:8%) diets were used. Single and combined exposures of Poly I:C (viral mimetic: 5 mg/kg body weight) and Lipopolysaccharide (LPS; bacterial endotoxin: 0.3 mg/kg body weight) were injected to both HP and LP pups at postnatal days (PND) 3 and 9 respectively, creating eight groups: HP (control); HP+Poly I:C; HP+LPS; HP+Poly I:C+LPS; LP; LP+Poly I:C; LP+LPS; LP+Poly I:C+LPS (multi-hit). The effects of stressors on hippocampal cytoarchitecture and behavioral abilities were studied at PND 180. LP animals were found to be more vulnerable to immune stressors than HP animals and symptoms like neuronal damage, spine loss, downregulation of Egr 1 and Arc proteins, gliosis and behavioral deficits were maximum in the multi-hit group. Thus, from these findings it is outlined that cellular and behavioral changes that occur following multi-hit exposure may predispose individuals to developing Schizophrenia-like pathologies during adulthood.

## Abstract (precis)

**Summary:** This study reports that exposure to perinatal multi-hit stress (protein malnourishment and immune stress) causes changes in the hippocampal cells alongside behavioral deficits which are also observed in Schizophrenic condition.

## INTRODUCTION

Schizophrenia is a chronic mental health condition which is accelerating in the present population at an alarming rate. This neurological disorder is characterized by a compromised mental and social health, prominently visible during late adulthood and continuing to worsen with age (Girardi et al., 2014; Moyer et al., 2015). Schizophrenia has been variously linked with a stressed early life environment, especially when more than one type of stressor, like nutritional deficiencies, viral and bacterial infections, trauma and social abuse (Opendak et al., 2017; Sarkar et al., 2019; Teissier et al., 2020) are encountered simultaneously (multi-hit), the development of schizophrenic symptoms are accelerated many folds in affected individuals.

In the present scenario, the concept of the multi-hit arises with the fact that the early life environment is prone to many stressors and the probability of more than one type of stressor acting simultaneously in a developing brain is very high. Stress-induced changes in the nervous system are characterized by neuronal degeneration, spine loss, gliosis and glial degeneration. Furthermore, stressors that are responsible for decrease in spine density also cause synaptic loss and neuronal death (Bennett, 2011; Li et al., 2015). Such synaptic remodulations can be correlated with neuronal loss, alongside changes in the expression pattern of early growth-response gene (Chang et al., 2017; Kim et al., 2018; Berdenis van Berlekom et al., 2019). Decreased spine count along with neurodegeneration, glial activation and behavioral impairments are common features of Schizophrenic conditions which further can be correlated with a stressed early period of life (Radley et al., 2013; Duman and Duman, 2015; Mizutani et al., 2019). Early growth response gene Egr 1/*Zif268* is an immediate early gene which regulates the expression of other genes like Platelet derived growth factor-A (PDGF-A), transforming growth factor-β1 (TGF-β), N-methyl-D-aspartate receptor (NMDA), activity regulated cytoskeleton (Arc) and nerve growth factor receptor (NGFR) (Duclot and Kabbaj, 2017; Marballi and Gallitano, 2018; Aliperti et al., 2019; Sun et al., 2019). Among the target genes, Arc is a plasticity associated effector gene that is responsible for maintaining brain plasticity through phosphorylation and expansion of the cytoskeleton during memory consolidation. Any changes in the synaptic condition are said to change the expression pattern of the Arc protein, which in turn affect the neuronal connectivity (Sun et al., 2019).

Early life stressors which also includes exposure to LPS and Poly I:C are reported to activate microglia and astrocytes (Patro et al., 2013; Nagayach et al., 2014; Sharma et al., 2016; Jäkel and Dimou, 2017). Neuronal or glial degeneration which occurs due to early life stress exposure is observed to vigorously activate microglia (Savage et al., 2019). Such activation of microglia is marked by an upregulation of MHC II protein on their cell surface and persistent uncontrolled activation further leads to chronic inflammation in the brain (Paolicelli and Ferretti, 2017; Askew and Nicola, 2018; Schetters et al., 2018). Astrocytes, on the other hand, maintain the synapses and brain homeostasis by effective uptake of neurotransmitter from the synaptic cleft and preventing glutamate excitotoxicity (Liu et al., 2018; Kim et al., 2019). Astrocytes are also affected by stress and neuronal death, which cause astrogliosis or astrocytic degeneration (Patro et al., 2015; Sharma et al., 2016; Naskar and Chattarji, 2019). All these changes in the cellular conditions of the brain are responsible for behavioral and cognitive impairments like anxiety, hyperactivity, depression and memory deficits (Keverne et al., 2015; McEwen et al., 2016) which are the hallmark of an important neurological disorder, namely Schizophrenia (Dillon et al., 2013; Trojsi et al., 2018; Sarkar et al., 2019). However, the correlation between multiple stressors and the development of Schizophrenic symptoms is still under investigation and the present study has been designed to investigate, how the cumulative action of multiple early life stress or hits would affect the cellular and synaptic conditions of brain, further affecting the behavioral abilities.

## RESULTS

### Pronounced neuronal degeneration and CA layer dystrophy observed through Golgi studies in multi-hit animals (LP+Poly I:C+LPS)

**Fig. 1.**
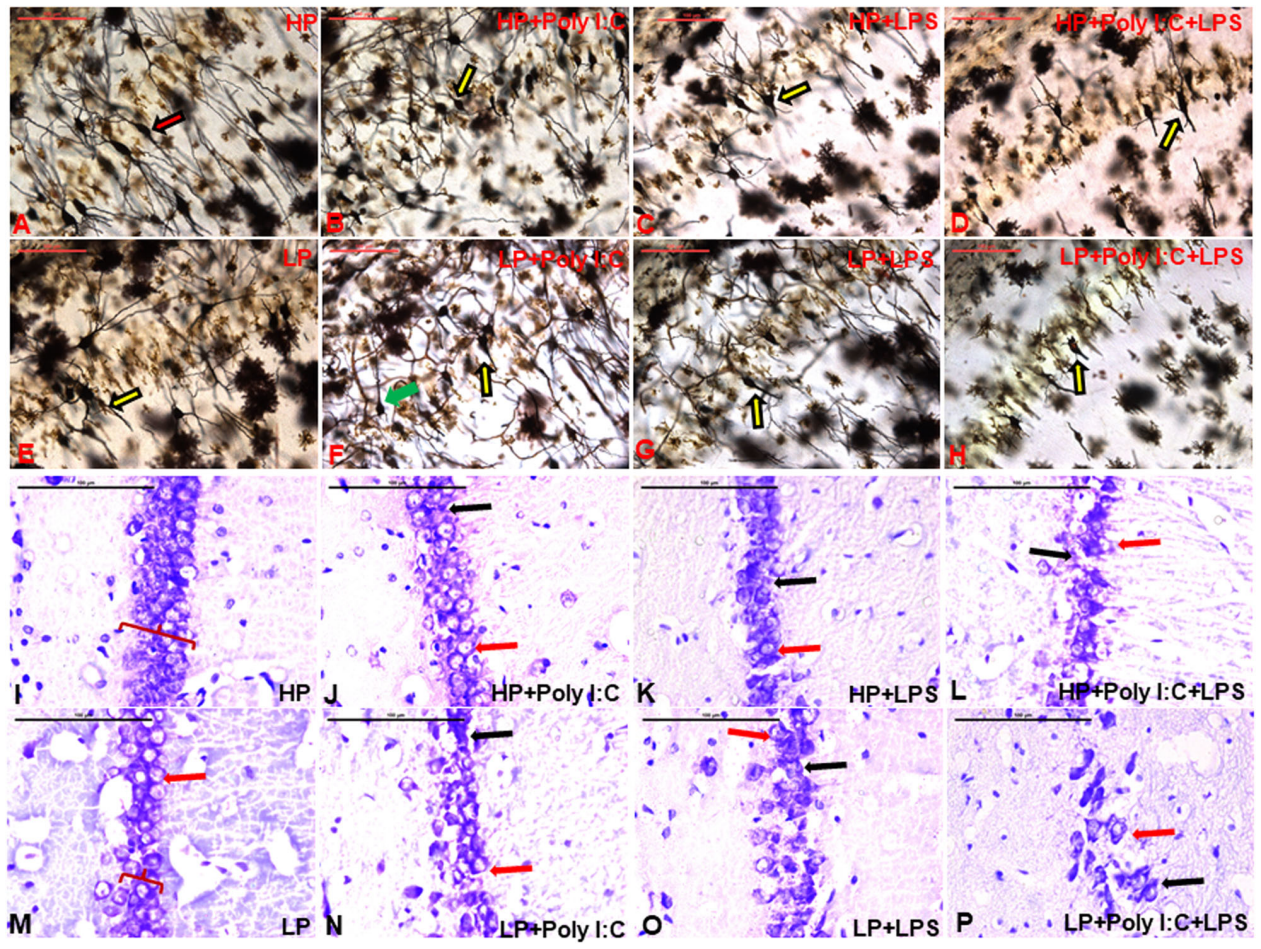
**Golgi and CV-stained images of CA pyramidal neurons showing stress related neuronal damage and an increase in degenerating cells.** From the Golgi-stained images, it is seen that the LP animals had poor density of neurons and less dendritic branching (yellow arrow, E) compared to densely arborized HP neurons (red arrow, A). Poly I:C and LPS exposure to both HP and LP animals deteriorated neuronal morphology in HP+Poly I:C (B), HP+LPS (C), HP+Poly I:C+LPS (D), LP+Poly I:C (F) and LP+LPS (G) groups, with maximum architecturally damaged neurons seen in multi-hit group (LP+Poly I:C+LPS, H). Additionally, misaligned and confined neurons (green arrow) were spotted in LP+Poly I:C group (*n*=6 slides from six different animals/group, scale bar=100 µm). In the CV-stained images, the CA region of the hippocampus was seen to be populated with healthy neurons in HP animals (I) while apoptotic bodies (red arrow) and decreased CA layer thickness was prominent in LP animals (M). Following single and combined exposure of Poly I:C and LPS to LP animals, there was further hype in layer degradation, apoptotic bodies (red arrows) and chromatolysis (black arrows) (N,O and P). Similar exposures given to HP animals (J,K and L) caused much less damage in HP animals in comparison to their LP counterparts (*n*=6 slides from six different animals/group, scale bar: 100 µm).

The hippocampal CA regions of HP control group rats demonstrated numerous healthy pyramidal neurons (red arrow), with long and branched dendritic processes (Fig. 1A). Either Poly I:C or LPS exposure to HP animals decreased healthy neuronal population in the CA layers, and many neurons without secondary and tertiary dendritic branches were observed in HP+Poly I:C (Fig. 1B) and HP+LPS (Fig. 1C) groups (yellow arrows). Combined exposure of both Poly I:C and LPS to HP animals resulted in appreciable changes in the neuronal morphology and they appeared stunted, with only primary dendrites and limited further branched processes (yellow arrow, Fig. 1D). LP alone animals also had morphologically deteriorated pyramidal neurons in the CA layers (Fig. 1E) with a similar appearance in both Poly I:C or LPS exposed LP groups. However in both LP+Poly I:C and LP+LPS groups, the neurons were found to be mostly disoriented with haphazardly arranged dendritic branches, (green arrow, Fig. 1F,G). Synergistic exposure of Poly I:C and LPS to LP animals (multi-hit), resulted in a severe decrease in CA neuron population (Fig. 1H) when compared to rest of the groups and the surviving neurons of this group were seen to possess only short primary dendrites with complete loss of dendritic arborization, dendritic spines and synaptic connections, indicating extensive neurodegeneration upon multi-hit exposure.

### CV staining further confirmed layer dystrophy and neuronal degeneration

From the cresyl violet stained images, it could be visualized that in HP control group, the CA layer was evenly packed with healthy neurons. The neuronal soma was prominent with no membrane blabbing, disruption or granulation (Fig. 1I). On the other hand, pyramidal layer thickness was reduced in LP alone group, consisting of numerous degenerating cells (red arrow, Fig. 1M). Single exposure of either Poly I:C or LPS to both HP control and LP alone animals led to cellular dystrophy along with chromatolysis (black arrows), membrane dystrophy, cellular shrinkage and apoptotic bodies (red arrows) visible in the CA layer of HP+Poly I:C, HP+LPS, LP+Poly I:C and LP+LPS animals (Fig. 1J,K,N,O). However, severe cellular degeneration and CA layer dystrophy with a very limited number of cells were seen in LP multi-hit group (LP+Poly I:C+LPS, Fig. 1P). Additionally, void areas, along with prominent apoptotic bodies and pyknotic neurons, were also visible in LP+Poly I:C+LPS group. Also, when similar exposure of Poly I:C+LPS was given to HP animals, the effect was comparatively less degenerative, which could be because of the endurance capacity of HP animals (Fig. 1L).

### Vigorous increase in degenerating neurons and decrease in spine density on multi-hit exposure (LP+Poly I:C+LPS)

**Fig. 2.**
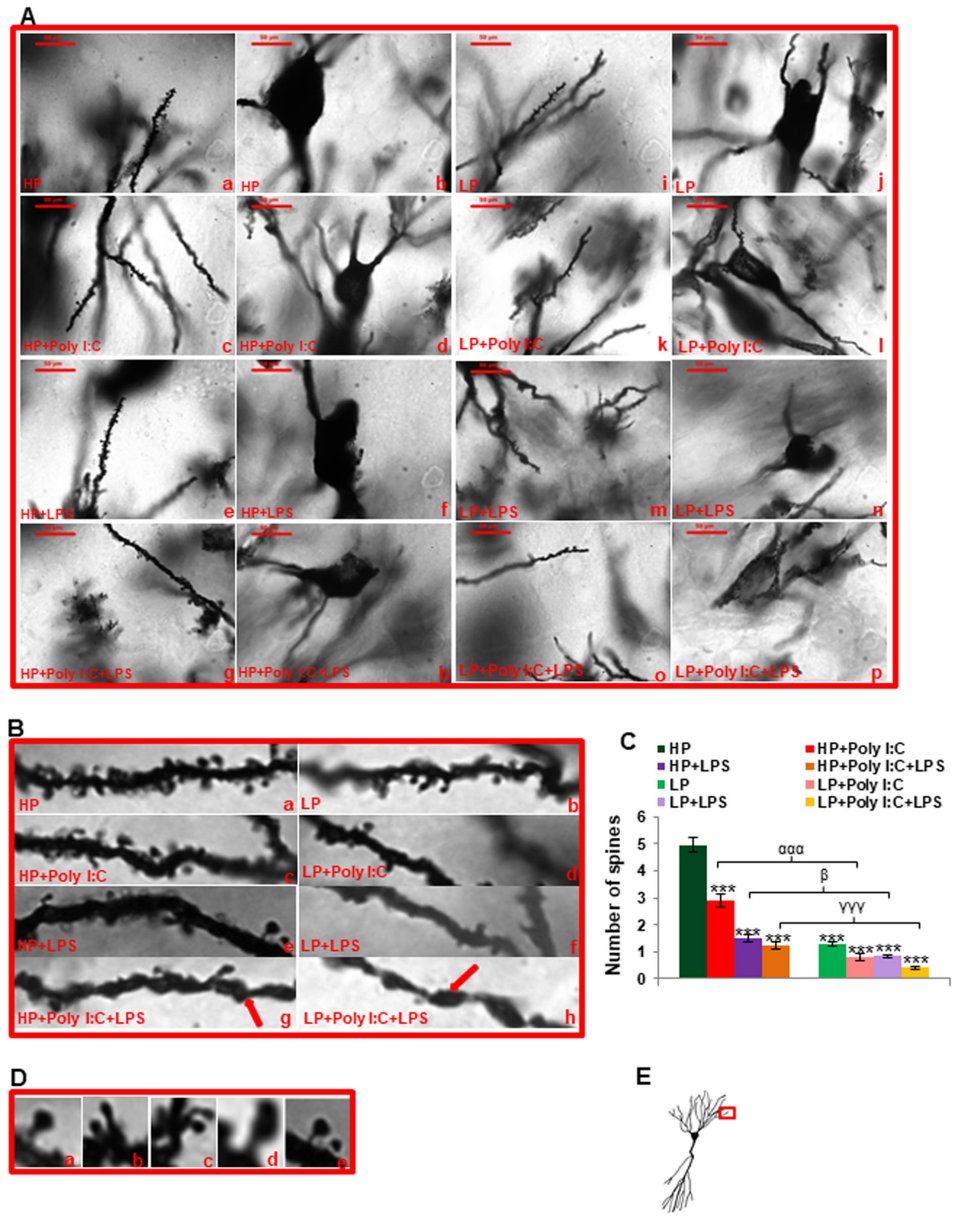
**Golgi images and histogram showing changes in dendritic arbor and spine density.** HP control animals had morphologically preserved somal and dendritic structures with evenly distributed healthy spines (Aa,b,Ba,Da,) in the secondary basal dendrites (E). LP alone group on the other hand had crooked dendrites (Ai), uneven soma (Aj) and unhealthy spines (Db,c,d,e) in their dendrites (Bb). Single and combined hit of Poly I:C and LPS to both HP and LP animals degraded the overall morphology of neurons and spines in HP+Poly I:C (Ac,d,Bc), HP+LPS (Ae,f,Be), HP+Poly I:C+LPS (Ag,h,Bg), LP+Poly I:C (Ak,l,Bd), LP+LPS (Am,n,Bf) and LP+Poly I:C+LPS (Ao,p,Bh) groups. LP+Poly I:C+LPS animals were seen to have maximum damaged neurons, with dendritic varicosities observed in the dendrites (red arrow, Bg,h), (*n*=6 slides from six different animals per group, scale bar: 100 µm). The histogram depicting spine density (C) demonstrates Poly I:C and LPS mediated low density of spines indicating their loss in both HP and LP animals, which however was maximum in LP+Poly I:C+LPS animals (*n*=108 dendrites from 36 different neurons, six slides from six different animals per group), values are expressed as mean±s.e.m.; ***P≤0.001 with respect to controls; ^###^*P*≤0.001 with respect to LP alone group; ^ααα^*P*≤0.001 with respect to HP+Poly I:C and LP+Poly I:C; ^β^*P*≤0.05 with respect to HP+LPS and LP+LPS; ^γγγ^*P*≤0.001 with respect to HP+Poly I:C+LPS and LP+Poly I:C+LPS.

In addition to the neuronal changes, spine density in the basal dendrites (Fig. 2E) was also found to be severely affected by exposure to early life stressors. In HP control animals, CA pyramidal neurons had well-defined soma (Fig. 2Ab) along with more or less evenly distributed spines in secondary basal dendrites (Fig. 2Aa). Moreover, the spine morphology (Fig. 2Ba) was figured out to be mostly mushroom shaped (Fig. 2Da), that are considered to be healthy and functional. In contrast to this, the LP alone animals had constricted soma (Fig. 2Aj) with significantly reduced number and uneven distribution of spines on their secondary basal dendrites with very few mushroom shaped spines (Fig. 2Ai,Bb). On single administration of Poly I:C or LPS to the HP animals, spine density was found to be decreased (Fig. 2Ac,e). However, a drastic decrease in spine density was observed in LP+Poly I:C and LP+LPS treated groups in comparison to LP alone animals (Fig. 2Ak,m). Additionally, morphology of soma was also found to be distorted in Poly I:C and LPS treated animals (Fig. 2Ad,f,l,n). Again, most of the spines in dendritic branches were of immature type (filopodia shaped, stubby, disoriented or long necked Fig. 2Dc,b,d,e) in HP+Poly I:C, HP+LPS, LP+Poly I:C and LP+LPS groups (Fig. 2Bc,d,e,f). Combined exposure of Poly I:C and LPS to LP animals resulted in further severe changes in the spine density with only few spines visible on the dendrites of LP multi-hit group when compared to rest of the treated groups (Fig. 2Ao). Also, the neuronal soma observed was invariably disintegrated, suggesting chromatolysis (Fig. 2Ap). However, spine and somal conditions were better in HP+Poly I:C+LPS animals (Fig. 2Ag, h) when compared to the LP multi-hit counterparts. Varicosities (Fig. 2Bg,h, red arrows) were observed in the dendrites of both HP+Poly I:C+LPS and LP+Poly I:C+LPS animals which might again be linked to spine engulfment, resulting in minimal number of spines in the dendritic branches of such multi-hit animals (Fig. 2Bg,h).

The result vide supra, depicting loss of dendritic spines in the treated groups can also be figured out from the histogram (Fig. 2C), with highest spine density in HP control group which decreased significantly following Poly I:C and LPS treatment (interaction of treatments within HP groups) either singularly i.e. HP+Poly I:C [*F*(3,428)=2, *P*≤0.001], HP+LPS [*F*(3,428)=3.4, *P*≤0.001] or in combination i.e. HP+Poly I:C+LPS [*F*(3,428)=3.74, *P*≤0.001]. Conditions were much more severe in LP alone group [*F*(1,642)=3.6, *P*≤0.001] than in the corresponding HP control group (impact of diet) which further decreased drastically on Poly I:C or LPS treatment to LP animals with a significant difference between HP and LP+Poly I:C and LP+LPS groups [*F*(7,856)=3.8, *P*≤0.001; *F*(7,856)=4, *P*≤0.001]. The spine density was minimal in LP multi-hit [LP+Poly I:C+LPS, *F*(7,856)=4.5, *P*≤0.001, t=20.2] group when compared to rest of the treated groups. Also when compared amongst groups, the LP animals reacted vigorously to any further stress in terms of spine loss showing impact of LP diet when compared to HP. Significant differences were found between LP+Poly I:C and HP+Poly I:C (*F*(1,642)=2.1, *P*≤0.001), LP+LPS and HP+LPS (*F*(1,642)=0.66, *P*≤0.05), LP+Poly I:C+LPS and HP+Poly I:C+LPS (*F*(1,642)=0.8, *P*≤0.001) groups.

### Multi-hit exposure (LP+Poly I:C+LPS) led to vigorous downregulation of Egr 1 protein in the hippocampus

**Fig. 3.**
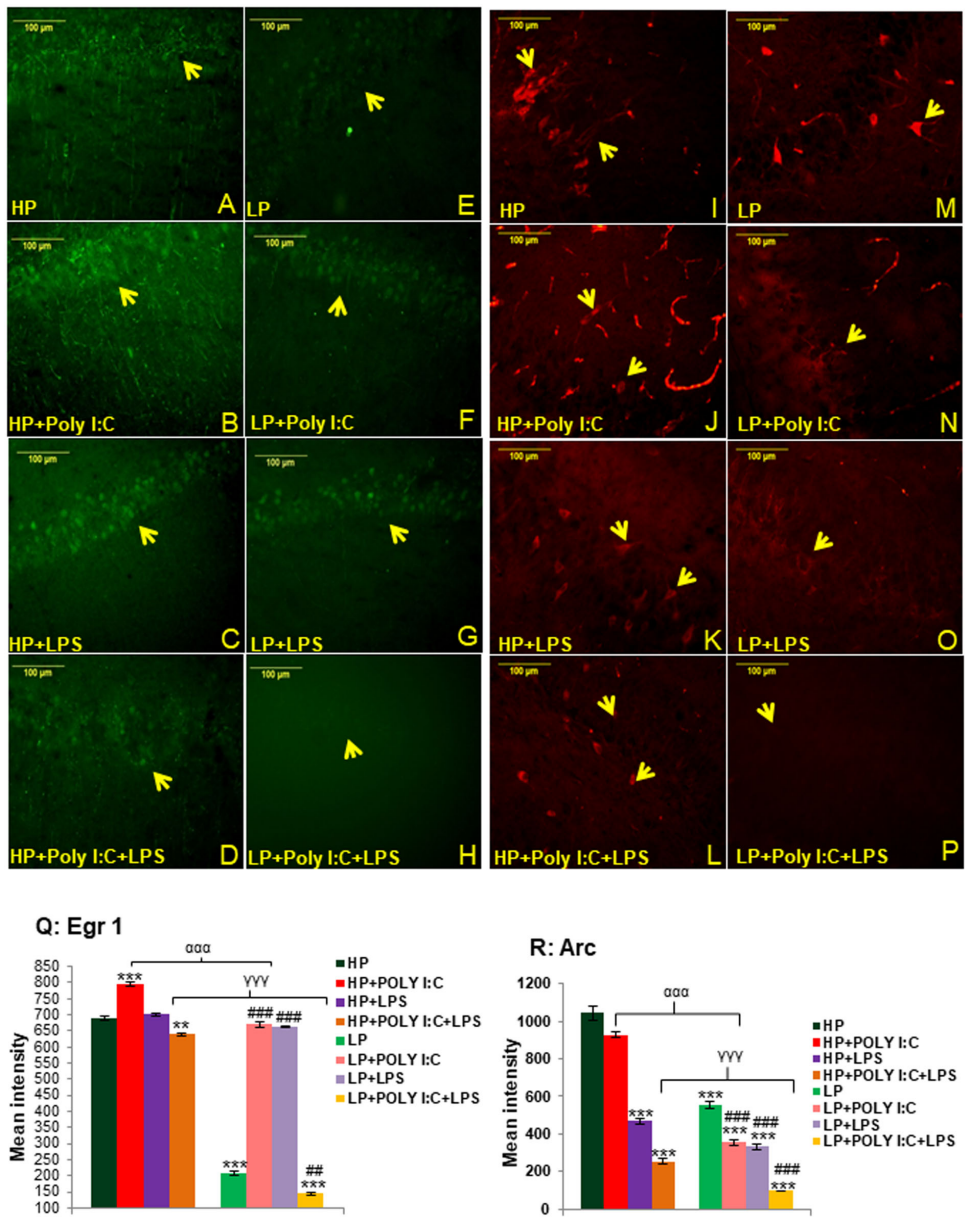
**Egr 1 and ARC expression in CA neurons showing stress mediated changes.** LP alone group had downregulated Egr1 expression (E), when compared to HP control (A), while single exposure of either Poly I:C or LPS upregulated Egr1 expression in both HP (B,C) and LP (F,G) groups. On combined exposure of Poly I:C and LPS, both HP+Poly I:C+LPS and LP+PolyI:C+LPS groups showed very low expression of Egr 1 protein (D,H), with minimal expression observed in LP multi-hit group. Arrows showing cells expressing Egr 1 protein, (*n*=6 slides from six different animals/group, scale bar: 100 µm). The quantitative data (Q) also depicts result that tally with the immunofluorescence images of Egr 1 protein, with minimum Egr 1 protein expression detected in LP+Poly I:C+LPS group (*n*=36 images, six slides from six different animals per group), values are expressed as mean±s.e.m.; ***P≤0.001,*P≤0.05 with respect to controls; ^###^*P*≤0.001,^#^*P*≤0.05 with respect to LP alone group; ^ααα^*P*≤0.001 with respect to HP+Poly I:C and LP+Poly I:C; ^γγγ^*P*≤0.001 with respect to HP+Poly I:C+LPS and LP+Poly I:C+LPS. The acquired CA images revealed that HP control had strongly labeled Arc expressing cells (I) in contrast to the mild labeling in LP alone group (M). Single exposure of Poly I:C (J,N) or LPS (K,O) decreased Arc protein expression in both HP and LP animals. Also, the downregulation was further severe when stressors were combined (L,P) with minimal expression in LP+Poly I:C+LPS group (P). Arrows showing Arc expressing cells, (*n*=6 slides from six different animals per group, scale bar: 100 µm). The bar graph depicting mean intensity of Arc protein (R) also demonstrates similar trend of Arc expression in treated groups with LP+Poly I:C+LPS group showing minimum protein expression (*n*=36 images, six slides from six different animals per group), values are expressed as mean±s.e.m.; ***P≤0.001with respect to controls; ^###^*P*≤0.001 with respect to LP alone group; ^ααα^*P*≤0.001 with respect to HP+Poly I:C and LP+Poly I:C; ^γγγ^*P*≤0.001 with respect to HP+Poly I:C+LPS and LP+Poly I:C+LPS.

In HP control, the Egr1 protein (arrow) was well expressed in CA neurons (Fig. 3A) and on LP stress, it was observed to be downregulated (Fig. 3E). Single hit of either Poly I:C or LPS to both HP and LP animals triggered Egr1 protein upregulation as observed in the preparations of HP+Poly I:C (Fig. 3B), HP+LPS (Fig. 3C), LP+Poly I:C (Fig. 3F) and LP+LPS (Fig. 3G) groups. However, when both Poly I:C and LPS were exposed simultaneously to HP and LP animals, both HP+Poly I:C+LPS and LP+Poly I:C+LPS (Fig. 3D,H) groups showed downregulation of Egr1 protein, with comparably more drastic reduction in LP+Poly I:C+LPS (multi-hit) group showing a very faint Egr1 expression in the CA neurons.

The above findings were supported by the quantitative data (Fig. 3Q) and interaction within treatments in respective HP and LP groups showed a significant downregulation of Egr1 protein in HP+Poly I:C+LPS group when [*F*_(3,140)_=50, *P*≤0.01] compared to the HP control and in LP multi-hit group [*F*_(3,140)_=482, *P*≤0.01] compared to LP alone group respectively. Groupwise interaction also indicated, lowest intensity of protein expression in LP+Poly I:C+LPS group [*F*_(7,280)_=546, *P*≤0.001, t=64.07]. However, single exposure of either Poly I:C or LPS to both HP as well as LP animals significantly hyped Egr1 expression in HP+Poly I:C group when compared to HP control [*F*_(3,140)_=104.22, *P*≤0.001] and in LP+Poly I:C and LP+LPS groups when compared to LP alone group [*F*_(3,140)_=462.3, *P*≤0.001; *F*_(3,140)_=456.9, *P*≤0.001]_._ Additionally, impact of diets upon treatments was seen as significant difference between HP versus LP, HP+PolyI:C versus LP+Poly I:C and HP+Poly I:C+LPS versus LP+Poly I:C+LPS groups [*F*_(1,210)_=482.9, *P*≤0.001; *F*_(1,210)_=124.9, *P*≤0.001; *F*_(1,210)_=496, *P*≤0.001], showing the vulnerability of LP group animals to any further immune stress.

### Minimal expression of Arc protein is seen in the multi-hit group (LP+Poly I:C+LPS)

From the microscopic images of anti-Arc labeled hippocampal areas, it could be seen that HP control group had intensely labeled Arc expressing cells (yellow arrows) in the CA regions of hippocampus (Fig. 3I). However, the Arc expression was observed to be downregulated in LP alone group (Fig. 3M). On exposure to either Poly I:C or LPS to HP and LP animals, the Arc expressing cells were found to be decreased in all the groups i.e., HP+Poly I:C (Fig. 3J), HP+LPS (Fig. 3K), LP+Poly I:C (Fig. 3N) and LP+LPS (Fig. 3O), when compared to non-immune stressed HP and LP groups. Furthermore, when both Poly I:C and LPS were simultaneously administered to HP and LP animals, the LP+Poly I:C+LPS group reacted more vigorously than HP+Poly I:C+LPS (Fig. 3L) group with minimal Arc expression visible in LP+Poly I:C+LPS group (Fig. 3P). However, the inter group comparison clearly indicated that, LP groups showed diffused Arc expression in CA layers as compared to their HP counterparts.

The histogram representing mean Arc fluorescence intensity (Fig. 3R) demonstrate maximum intensity in HP control and a significant downregulation in LP alone group [*F*_(1,210)_=488, P*P*≤0.001, impact of diet]. On single or combined exposure of Poly I:C and LPS to HP and LP animals, the mean intensity of Arc protein decreased significantly in HP+Poly I:C [*F*_(3,140)_=115, *P*≤0.05], HP+LPS [*F*_(3,140)_=575.07, *P*≤0.001], HP+Poly I:C+LPS [*F*_(3,140)_=789.33, *P*≤0.001] when compared to HP control and in LP+Poly I:C [*F*_(3,140)_=202.34, *P*≤0.001], LP+LPS [*F*_(3,140)_=222, *P*≤0.001] and LP+Poly I:C+LPS [*F*_(3,140)_=457.2, *P*≤0.001] groups when compared to LP alone group as well as HP control [*F*_(7,280)_=690, *P*≤0.001; *F*_(7,280)_=710, *P*≤0.001; *F*_(7,280)_=945, *P*≤0.001], respectively. Additionally, LP+Poly I:C+LPS rats showed lowest Arc intensity (t=33.3) and significant impact of LP diet on treatment was seen as significant difference between HP+Poly I:C versus LP+Poly I:C [*F*_(1,210)_=595, *P*≤0.001] and HP+Poly I:C+LPS versus LP+Poly I:C+LPS (*F*_(1,210)_=155.2, *P*≤0.001) groups.

### Iba1 labeling revealed stress-induced microgliosis

**Fig. 4.**
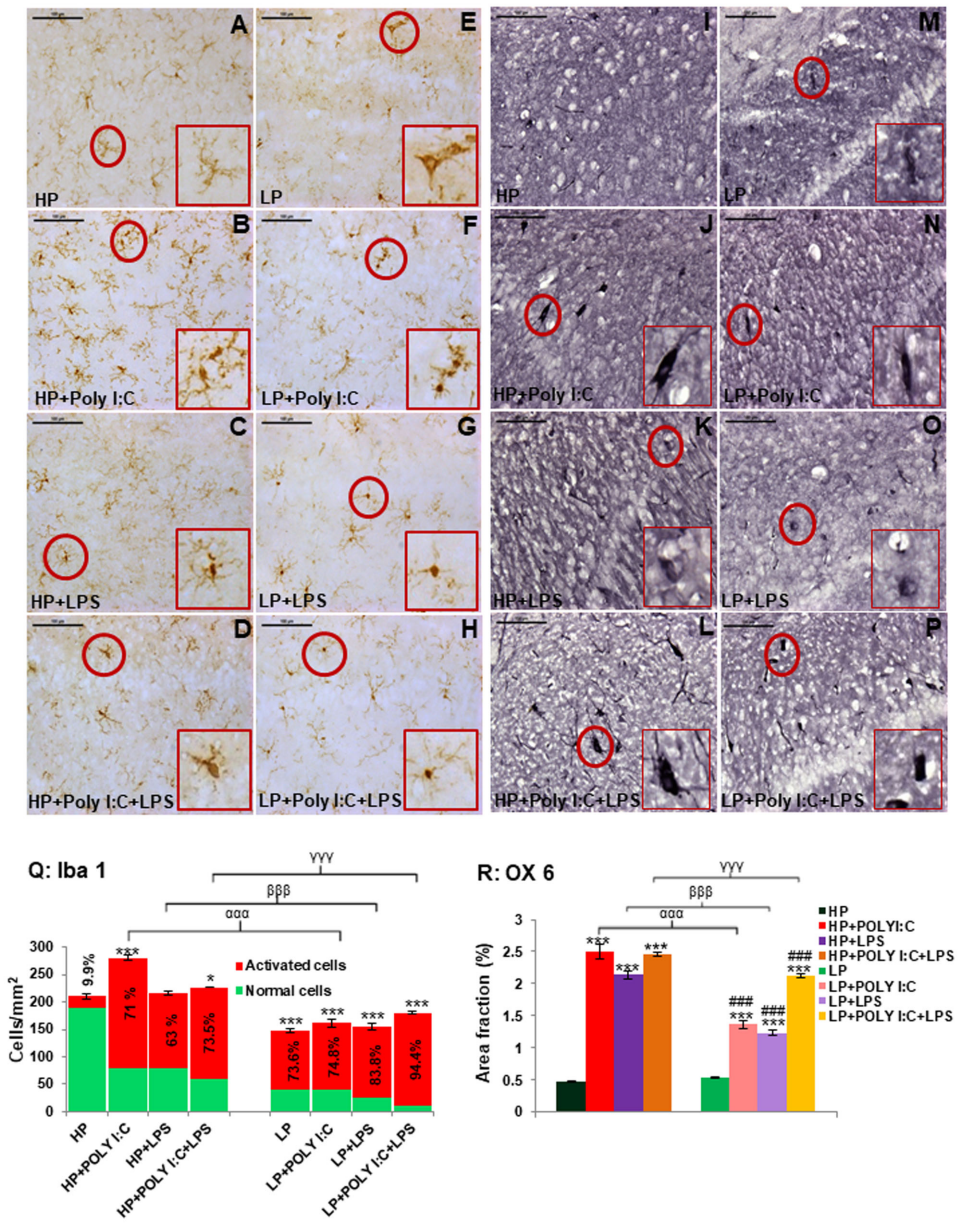
**Iba 1 and OX 6 labeled immunohistochemical images and stacked graph showing stress-related changes in microglial population.** In HP controls the resting ramified microglia are seen scattered throughout the Iba 1 labeled image area (A), whereas in LP groups the microglia are limited in number, with varying activated states (E). Poly I:C and LPS exposure increased activated microglial population in both HP and LP stressed groups (B,C,D,F,G,H), with vigorous hype in activated cells spotted in LP+Poly I:C+LPS group (H), (*n*=6 slides from six different animals per group, scale bar: 100 µm). The quantitative data representing the total as well as activated hippocampal microglial population in different groups (Q), confirms the results shown above (*n*=108 images, six slides from six different animals per group), values are expressed as mean±s.e.m.; ***P≤0.001 with respect to controls; ^###^*P*≤0.001 with respect to LP alone group. From the OX 6 labeled images it is evident that both single and combined exposure of Poly I:C and LPS to HP and LP animals hyped MHC II expression in the hippocampus of the stressed animals when compared to HP control (I) and LP alone groups (M). HP+Poly I:C (J), HP+LPS (K) and HP+Poly I:C+LPS groups (L) were seen to contain intensely labeled OX 6 positive cells around the CA layer, which was comparatively less prominent in LP groups (N-P), (*n*=6 slides from six different animals per group, scale bar: 100 µm). From the quantification data (R) it was seen that, MHC II expression increased in both HP and LP animals, consequent upon Poly I:C and LPS treatment. LP groups however showed comparatively less MHC II expression when compared to similarly treated HP groups. (*n*=108 images, six slides from six different animals per group), values are expressed as mean±s.e.m.; ***P≤0.001 with respect to controls; ^###^*P*≤0.001 with respect to LP alone group; ^ααα^*P*≤0.001 with respect to HP+Poly I:C and LP+Poly I:C; ^βββ^*P*≤0.05 with respect to HP+LPS and LP+LPS; ^γγγ^*P*≤0.001 with respect to HP+Poly I:C+LPS and LP+Poly I:C+LPS.

In HP control animals, resting ramified microglia were seen in the hippocampus (Fig. 4A). Poly I:C and LPS exposure to HP animals either individually or in combination, led to an increase in density of microglial population including varying activated states with fragmented processes in the hippocampus of HP+Poly I:C, HP+LPS and HP+Poly I:C+LPS animals when compared to HP control rats (Fig. 4B,C,D). In LP alone as well as LP+Poly I:C and LP+LPS groups, the overall microglial cells were limited in number, while percentage of activated cells increased, as compared to HP control (Fig. 4E,F,G). However, a drastic increase in activated microglial cell population was seen when both Poly I:C and LPS were exposed to LP animals (Fig. 4H).

The results vide supra were depicted through the stacked bar graph (Fig. 4Q), representing percent of the activated microglia out of the total Iba 1 positive microglial population. The number of activated microglia was very low in HP control (9.9%) and although all the LP groups had significantly low total microglial population, the number of activated cells increased significantly as compared to HP control [*F*_(7,856)_=49, *P*≤0.001; *F*_(7,856)_=56, *P*≤0.001; *F*_(7,856)_=31, *P*≤0.001, groupwise comparison]. On single or combined exposure of Poly I:C and LPS to HP and LP animals, there was an increase in both the total number as well as the percentage of activated microglia. Interaction between treatments within HP and LP groups led to significant difference between HP versus HP+Poly I:C, HP versus HP+Poly I:C+LPS [*F*_(3,428)_=69, *P*≤0.001; *F*_(3,428)_=16, *P*≤0.05] and LP versus LP+Poly I:C+LPS [*F*_(3,428)_=32, *P*≤0.001]. However, in LP multi-hit group (LP+Poly I:C+LPS) most of the microglia were in their activated states (94.4%) when compared to rest of the groups. Impact of diet over treatment was clearly evident as significantly lower total microglial population in LP, LP+Poly I:C, LP+LPS and LP+Poly I:C+LPS groups as compared to their HP counterparts [*F*_(1,642)_=63, *P*≤0.001; *F*_(1,642)_=118, *P*≤0.001; *F*_(1,642)_=62, *P*≤0.001; *F*_(1,642)_=47, *P*≤0.001].

### Stress dependent MHC II upregulation

The OX 6 immunolabelling for MHC II protein localization, revealed a few discreetly labeled cells in the CA layers of the LP alone group rats (Fig. 4M) in contrast to no clearly labeled cells in the HP control groups (Fig. 4I). Following Poly I:C or LPS exposure to HP and LP animals, MHC II protein expression was hyped when compared to HP control and LP alone group and OX 6 labeled cells were prominently seen in the hippocampal areas of HP+Poly I:C, HP+LPS, LP+Poly I:C and LP+LPS animals (Fig. 4J,K,N,O). Also, the combined exposure of Poly I:C and LPS to HP and LP animals further upregulated the MHC II protein with increase in both number and intensity of labeling of OX 6 positive cells in the hippocampus of HP+Poly I:C+LPS and LP+Poly I:C+LPS animals (Fig. 4L,P). However, the stressors exposed hype in positive cells were more prominent in HP groups when compared to the LP counterparts.

The above mentioned results also tallies with the mean area fraction data (Fig. 4R), according to which, HP and LP alone groups had least MHC II expression and on single and combined exposure of either Poly I:C or LPS, there was an upregulation of MHC II protein in all HP treated groups, i.e., HP+Poly I:C [*F*_(3,428)_=2, *P*≤0.001], HP+LPS [*F*_(3,428)_=1.6, *P*≤0.001] and HP+Poly I:C+LPS [*F*_(3,428)_=1.9, *P*≤0.001], when compared to HP control group (interaction of treatments within HP groups). Similar results were recorded in LP+Poly I:C [*F*_(7,856)_=0.8, *P*≤0.001; *F*_(3,428)_=0.83, *P*≤0.001], LP+LPS [*F*_(7,856)_=0.75, *P*≤0.001; *F*_(3,428)_=0.7, *P*≤0.001] and LP+Poly I:C+LPS [*F*_(7,856)_=1.6, *P*≤0.001; *F*_(3,428)_=1.5, *P*≤0.001] groups when compared to both HP control (groupwise comparison) and LP alone groups (interaction of treatments within LP groups) respectively. The differences were also found between HP+Poly I:C versus LP+Poly I:C [*F*_(1,642)_=1.1, *P*≤0.001], HP+LPS versus LP+LPS [*F*_(1,642)_=0.9, *P*≤0.001] and HP+Poly I:C+LPS versus LP+Poly I:C+LPS [*F*_(1,642)_=0.32, *P*≤0.001] groups indicating the impact of maternal protein deprivation. Lastly, the overall decreased MHC II area fraction in LP groups as compared to their respective HP counterparts, could be because of an overall decrease in total number of microglia (Fig. 4Q).

### Early-life stress and persistent astrogliosis

**Fig. 5.**
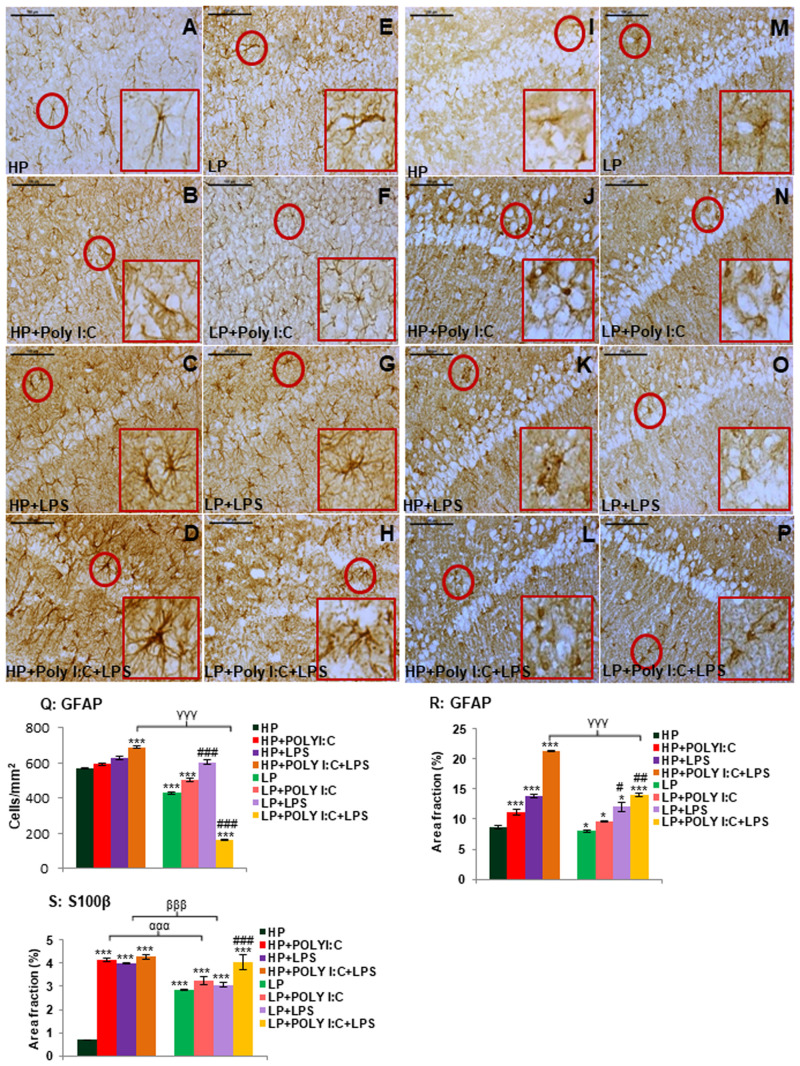
**GFAP and S100β labeled images and quantitative data demonstrates astrogliosis in stressed groups.** Healthy, star-shaped cells were spotted in GFAP labeled HP control (A), which decreased in LP alone group (E). Cells were observed to be hypertrophied in HP+Poly I:C (B), HP+LPS (C) and HP+Poly I:C+LPS (D) groups and in LP+Poly I:C (F) and LP+LPS (G) group, there was an increase in astrocytic population, which further decreased in multi-hit exposure (LP+Poly I:C+LPS, H), (*n*=6 slides from six different animals per group, scale bar: 100 µm). The result vide supra is also supported by the cell count and intensity measure (area fraction) graphs (Q and R), (*n*=108 images, six slides from six different animals per group), values are expressed as mean±s.e.m.; ***P≤0.001, *P≤0.001 with respect to controls; ^###^*P*≤0.001 with respect to LP alone group; ^ααα^*P*≤0.001 with respect to HP+Poly I:C and LP+Poly I:C; ^βββ^*P*≤0.05 with respect to HP+LPS and LP+LPS; ^γγγ^*P*≤0.001, ^γ^*P*≤0.05 with respect to HP+Poly I:C+LPS and LP+Poly I:C+LPS. While HP control had minimum S100β expression around CA layer (I), HP+Poly I:C, HP+LPS and HP+Poly I:C+LPS group had increased S100β expressing cells (J-L). LP alone animals also possessed more S100β expressing cells around CA layer (M), which further increased on Poly I:C and LPS treatment (N,O) and maximum S100β positive cells were found in LP+Poly I:C+LPS group (P). (*n*=6 slides from six different animals per group, scale bar: 100 µm). The quantitative data (S) also supports result observed in the above-mentioned images. (*n*=108 images, six slides from six different animals per group), values are expressed as mean±s.e.m.; ***P≤0.001, with respect to controls; ^###^*P*≤0.001 with respect to LP alone group; ^ααα^*P*≤0.001 with respect to HP+Poly I:C and LP+Poly I:C; ^βββ^*P*≤0.05 with respect to HP+LPS and LP+LPS.

Astroglial activation was a frequent observation in LP alone as well as following single or cumulative exposure of Poly I:C and LPS to both HP and LP groups, when compared to the HP controls. Activated astrocytes were clearly marked with phenotypic changes including prominent enlarged soma and darkly labeled thick processes, showing hypertrophy. In addition, the density of astrocytes was found to be increased in both HP+Poly I:C and HP+LPS groups when compared to HP controls (Fig. 5A-C). Further, on combined exposure of Poly I:C and LPS to HP animals, severe astrogliosis was observed with a vigorous increase in intensely GFAP labeled hypertrophied astrocytes, possessing much enlarged cell body and thick dense processes (Fig. 5D). The LP animals on the other hands showed fragmentation of the activated astrocytes (Fig. 5E). Singular administration of Poly I:C or LPS led to an increase in GFAP expressing cells in LP+Poly I:C and LP+LPS animals when compared to LP alone group (Fig. 5F,G). However, in LP+PolyI:C+LPS group animals, extreme cellular fragmentation was seen when compared to rest of the groups, accounting for an overall low astrocyte density in hippocampus (Fig. 5H).

The bar diagrams showing both the number of GFAP labeled cells and the area fraction confirms the qualitative results observed vide supra, indicating a significantly low astrocytic density and area fraction in LP groups as compared to the corresponding HP groups, significant difference was found between LP+Poly I:C+LPS and HP+Poly I:C+LPS groups [*F*_(1,642)_=529, *P*≤0.001; *F*_(1,642)_=7.2, *P*≤0.001] (Fig. 5Q,R). Impact of maternal diet was evident from low astrocytic number in LP alone group when compared to HP control [*F*_(1,642)_=142, *P*≤0.001]. On single or combined exposure of Poly I:C or LPS to HP animals, there was an increase in both cell number and GFAP expression in HP+Poly I:C [*F*_(3,428)_=3, *P*≤0.001], HP+LPS [*F*_(3,428)_=4.8, *P*≤0.001] and HP+Poly I:C+LPS [*F*_(3,428)_=409, *P*≤0.001; *F*_(3,428)_=5.3, *P*≤0.001] groups when compared to HP control (inter-group comparison within HP groups), suggesting astrogliosis. Although GFAP upregulation was clearly evident from the microscopic images following single or cumulative exposure to LP animals [*F*_(3,428)_=174, *P*≤0.001; *F*_(3,428)_=2, *P*≤0.05], the cell number remained significantly low in LP+Poly I:C+LPS group when compared to LP alone group because of the astrocytic fragmentation [*F*_(3,428)_=4, *P*≤0.001, interaction between treatment with in LP groups]. Significant difference was also found between HP versus LP+Poly I:C [*F*_(7,856)_=70, *P*≤0.001; *F*_(7,856)_=0.9, *P*≤0.001], HP versus LP+LPS [*F*_(7,856)_=3.3, *P*≤0.001] and HP versus LP+Poly I:C+LPS [*F*_(7,856)_=409, *P*≤0.001; *F*_(7,856)_=5, *P*≤0.001], groupwise comparison regarding cell number and area fraction, respectively.

### S100β upregulation and astrogliosis

Astrogliosis reported vide supra was further confirmed by S100β immunolabelling and its upregulation has been accepted as a marker of neurodegeneration. The results revealed strong S100β labeled astrocytes throughout the hippocampus in LP group preparation (Fig. 5M) in contrast to a very few S100β positive cells in the HP control (Fig. 5I). In addition, Poly I:C or LPS exposure either alone or in combination to HP rats resulted in a drastic increase in both the number of S100β positive astrocytes and its expression indicating severe astrogliosis (Fig. 5J-L). Most of such strongly S100β expressing cells were seen encircling the CA pyramidal neurons giving the appearance of a cluster. Although with Poly I:C or LPS treatment to LP rats as well, an increase in the S100β expression or the cells expressing this protein was evident but the difference among the LP groups was insignificant. This could be because of an overall decrease in astrocytic density (Fig. 5N,O,P).

These results were supported by the area fraction data (Fig. 5S) with a significant upregulation of protein expression in LP alone group when compared to HP control [*F*_(1,642)_=2.1, *P*≤0.001, impact of diet]. On single and combined exposure of Poly I:C and LPS to both HP and LP animals, there was a significant increase in mean area fraction of S100β protein in HP+Poly I:C [*F*_(3,428)_=3.4, *P*≤0.001], HP+LPS [*F*_(3,428)_=3.3, *P*≤0.001] and HP+Poly I:C+LPS [*F*_(3,428)_=3.5, *P*≤0.001] groups when compared to HP control and in LP+Poly I:C+LPS [*F*_(3,428)_=1.2, *P*≤0.001] group when compared to LP alone group respectively (interaction of treatment within HP and LP groups). Additionally, all the LP groups had hyped expression of S100β protein when compared to HP control [*F*_(7,856)_=3, *P*≤0.001; *F*_(7,856)_=2.6, *P*≤0.001, groupwise interaction] and impact of LP diet over treatment was seen as significant difference was found between HP versus LP [*F*_(1,642)_=2, *P*≤0.001], HP+Poly I:C versus LP+Poly I:C [*F*_(1,642)_=1, *P*≤0.001] and HP+LPS versus LP+LPS groups [*F*_(1,642)_=0.9, *P*≤0.001].

### Behavioral changes detected through EPM in treated groups

**Fig. 6.**
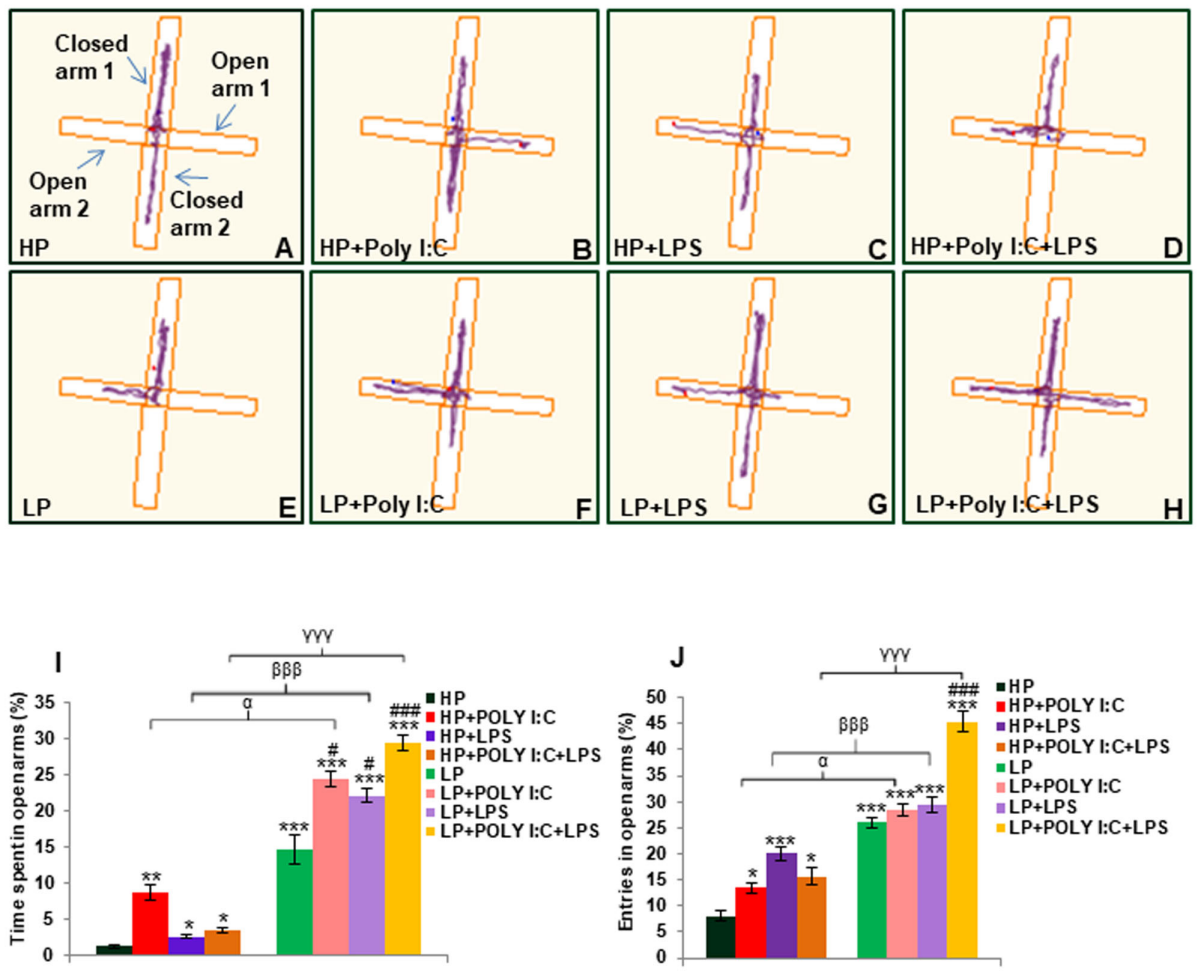
**EPM data showing low anxiety and hyperactivity like symptoms in LP+Poly I:C+LPS group.** The track records show normal exploratory behavior of rats in HP control group (A), whereas the stressed animals preferred to explore open arms over closed arms (B-H). Such behavioral deficit was highest in LP+Poly I:C+LPS group (H), (*n*=12 animals per group). From the bar diagrams representing percent time spent and number of entries (I,J), it was seen that the LP groups preferred open arms over closed arms thereby increasing the number of open arm entries and time spent in open arms. Significant difference in both the parameters was found in LP+Poly I:C+LPS group when compared to control, (*n*=12 animals per group), values are expressed as mean±s.e.m.; ***P≤0.001, **P≤0.005, with respect to controls; ^α^*P*≤0.05 with respect to HP+Poly I:C and LP+Poly I:C; ^βββ^*P*≤0.05 with respect to HP+LPS and LP+LPS; ^γγγ^*P*≤0.001, ^γ^*P*≤0.05 with respect to HP+Poly I:C+LPS and LP+Poly I:C+LPS.

From the track records, it was observed that the control animals explored the EPM while avoiding the edges of the open arms (Fig. 6A). On single and combined exposure of Poly I:C and LPS to HP animals, the treated animals started visiting the edges of the open arms, showing hyperactivity and low anxiety-like symptoms (Fig. 6B-D). On the other hand, LP animals lacked exploratory ability and were not able to differentiate between open and closed arms and showed higher preference for open arms (Fig. 6E). Poly I:C and LPS exposure either individually or in combination was found to deteriorate the behavior of LP animals and all the LP groups (LP+Poly I:C, LP+LPS and LP+Poly I:C+LPS) showed further high preference for open arms over closed arms (Fig. 6F-H), with maximum preference for open arms seen in LP+Poly I:C+LPS animals (Fig. 6H).

The above-mentioned observations were also evident from the histograms depicting percent time spent and entries in open arms (Fig. 6I,J). An increase in open arm activity in LP animals (both duration and entries) was seen when compared to HP controls, reflecting anti-anxiety like behavior [*F*_(1,66)_=5.9, *P*≤0.001; *F*_(1,66)_=5.9, *P*≤0.001, impact of diet]. Consequently upon single and combined exposure of Poly I:C and LPS to both HP and LP animals (HP+Poly I:C, HP+LPS, HP+Poly I:C+LPS, LP+Poly I:C, LP+LPS and LP+PolyI:C+LPS groups), there was a significant increase in time spent in open arms [*F*_(3,44)_=5.4, *P*≤0.01; *F*_(3,44)_=1, *P*≤0.05; *F*_(3,44)_=3.6, *P*≤0.05; *F*_(7,88)_=20, *P*≤0.001; *F*_(7,88)_=18, *P*≤0.001; *F*_(7,88)_=37, *P*≤0.001] and open arm entries [*F*_(3,44)_=10.4, *P*≤0.05; *F*_(3,44)_=15, *P*≤0.001; *F*_(3,44)_=12, *P*≤0.05; *F*_(3,44)_=7.5, *P*≤0.001; *F*_(7,88)_=19, *P*≤0.001; *F*_(7,88)_=20, *P*≤0.001; *F*_(7,88)_=28, *P*≤0.001] when compared to HP controls (treatments within HP groups and groupwise interaction). Again, interaction of treatments within LP groups showed significant difference between LP versus LP+Poly I:C [*F*_(3,44)_=9.8, *P*≤0.05], LP versus LP+LPS [*F*_(3,44)_=12, P*P*≤0.05] and LP versus LP+Poly I:C+LPS [*F*_(3,44)_=14, *P*≤0.001] groups regarding time spent in open arms and only between LP versus LP+Poly I:C+LPS [*F*_(3,44)_=19.4, *P*≤0.001], with respect to entries in open arms. However, maximum difference depicting severe behavioral changes was shown by LP+Poly I:C+LPS group (t=19.5, t=19.4) and lastly, impact of LP diet was seen as significant difference was found between HP+Poly I:C versus LP+Poly I:C [*F*_(1,66)_=15, *P*≤0.05; *F*_(1,66)_=14.8, *P*≤0.05]; HP+LPS versus LP+LPS [*F*_(1,66)_=19.5, *P*≤0.001; *F*_(1,66)_=9.3, *P*≤0.001] and HP+Poly I:C+LPS versus LP+Poly I:C+LPS [*F*_(1,66)_=26, *P*≤0.001; *F*_(1,66)_=29.4, *P*≤0.001] groups regarding time spent and number of entries in open arms respectively, suggesting a further build up in hyperactivity and low anxiety like symptoms in LP animals on immune stress exposure when compared to their HP counterparts.

### Hyperactivity and low anxiety-like symptoms were seen in the stressed groups

**Fig. 7.**
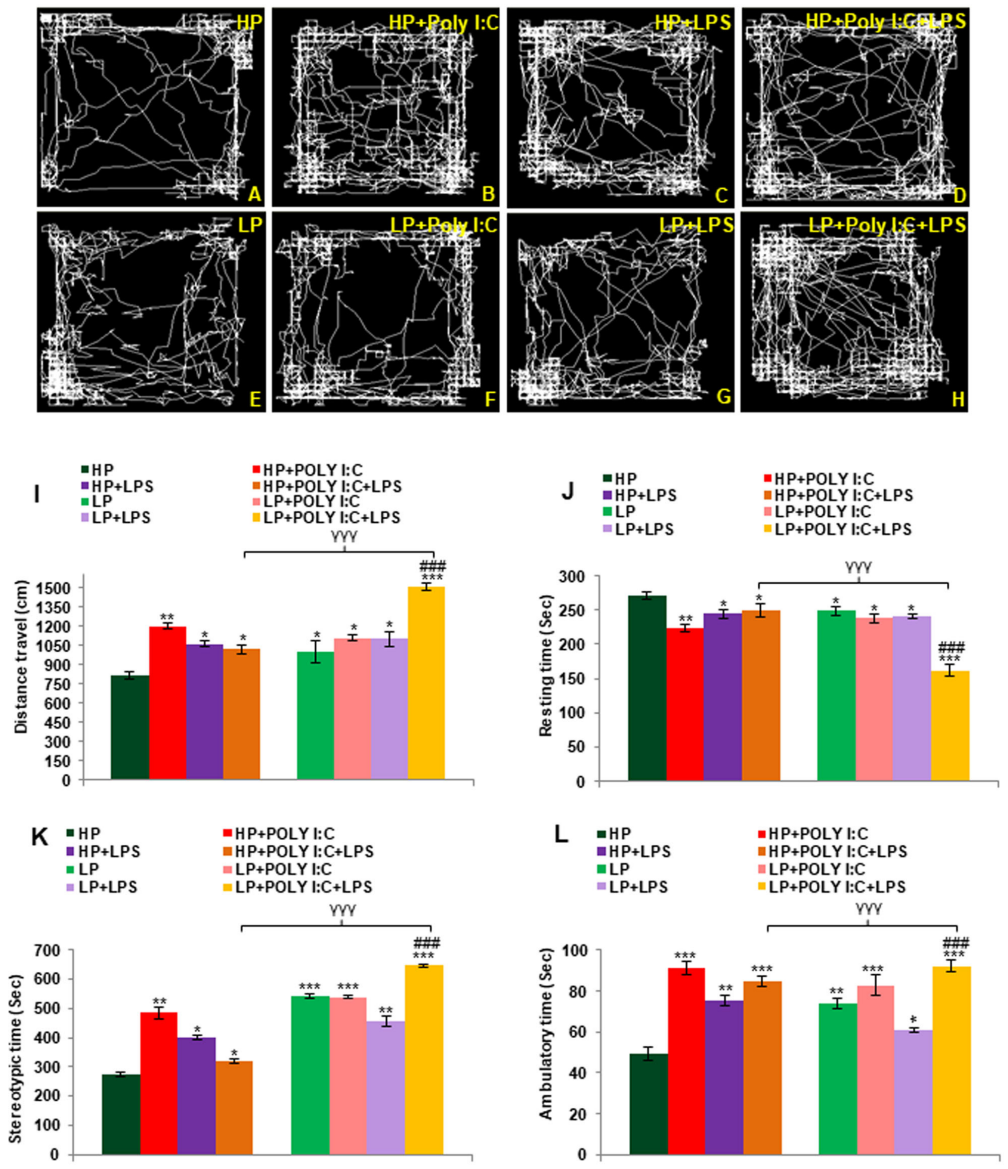
**OFT data showing low anxiety like symptoms in LP+Poly I:C+LPS group.** The track records indicate that the HP control animals securely explore the noble open arena of OFT (A). The HP+Poly I:C, HP+LPS and HP+Poly I:C+LPS group rats showed low anxiety and hyperactivity-like symptoms, travelling more distance and haphazardly exploring the central arena (B-D). LP alone animals also had increased distance travelled (E) and on immune stress, the animals belonging to LP+Poly I:C (F), LP+LPS (G) and LP+Poly I:C+LPS (H) showed further behavioral impairment with maximum deficit shown by LP+Poly I:C+LPS group (*n*=12 animals per group). From the distance travelled (I), resting time (J), stereotypic time (K) and ambulatory time (L) data, it is evident, that all the treated-group rats show increased distance travelled, stereotypic time and ambulatory time, with low resting time when compared to HP control. However, significant difference in all the parameters was found only between the HP control and the LP+Poly I:C+LPS groups (*n*=12 animals per group), values are expressed as mean±s.e.m.; ***P≤0.001, **P≤0.005, *P≤0.05 with respect to controls.

The OFT track records clearly depicted that HP control animals possessed normal explorative behavior (Fig. 7A), while the LP alone animals travelled quite often along the central zone than the HP control group, haphazardly exploring the open arena with complicated track patterns (Fig. 7E). On Poly I:C and LPS exposure to both HP and LP animals, low anxiety-like symptoms were observed in HP+Poly I:C (Fig. 7B), HP+LPS (Fig. 7C), LP+Poly I:C (Fig. 7F) and LP+LPS (Fig. 7G) groups. Whereas on multi-hit exposure severe behavioral deficits with haphazard track activity denoting severe hyperactivity or low anxiety like symptoms were visible in LP+Poly I:C+LPS group (Fig. 7H), as compared to HP+Poly I:C+LPS (Fig. 7D) and all the remaining groups respectively.

Parameters like distance travelled, resting time, stereotypic time and ambulatory time (Fig. 7I,J,K,L) were plotted as histograms and the data revealed that LP alone group animals possessed hyperactivity like behavior, showing effect of diet [*F*_(1,66)_=192, *P*≤0.05; *F*_(1,66)_=21, *P*≤0.05; *F*_(1,66)_=269, *P*≤0.001; *F*_(1,66)_=24, *P*≤0.01], which further increased on Poly I:C and LPS exposure to LP animals, with significant increase observed in distance travelled [*F*_(7,88)_=298, *P*≤0.05; *F*_(7,88)_=289, *P*≤0.05], stereotypic time [*F*_(7,88)_=265, *P*≤0.001; *F*_(7,88)_=183, *P*≤0.01], ambulatory time [*F*_(7,88)_=33*P*≤0.01; *F*_(7,88)_=11, *P*≤0.05] and decrease in resting time [*F*_(7,88)_=32, *P*≤0.05; *F*_(7,88)_=29, *P*≤0.05], when compared to both HP control (groupwise interaction) and LP alone groups respectively (interaction of treatment within LP groups). Such behavioral deficits were also seen in HP+Poly I:C [*F*_(7,88)_=388, *P*≤0.01; *F*_(7,88)_=47, *P*≤0.01; *F*_(7,88)_=211, *P*≤0.01; *F*_(7,88)_=41, *P*≤0.001], HP+LPS [*F*_(7,88)_=250, *P*≤0.05; *F*_(7,88)_=25, *P*≤0.05; *F*_(7,88)_=127, *P*≤0.05; *F*_(7,88)_=25, *P*≤0.01] and HP+Poly I:C+LPS [*F*_(7,88)_=208, *P*≤0.05; *F*_(7,88)_=20, *P*≤0.05; *F*_(7,88)_=45, *P*≤0.05; *F*_(7,88)_=35, *P*≤0.001] groups when compared to HP control (interaction of treatment within HP groups). However, behavioral deficits were highest in LP multi-hit i.e., LP+Poly I:C+LPS group with highest distance travelled [*F*_(7,88)_=699, *P*≤0.001; *F*_(3,44)_=507, *P*≤0.001, t=11.3], stereotypic time [*F*_(7,88)_=372, *P*≤0.001; *F*_(3,44)_=269, *P*≤0.001, t=23.8], ambulatory time [*F*_(7,88)_=42, *P*≤0.001; *F*_(3,44)_=24, *P*≤0.001, t=9.6] and minimal resting time [*F*_(7,88)_=108, *P*≤0.001; *F*_(3,44)_=87, *P*≤0.001, t=11.2] when compared with HP control and LP alone group. Additionally, impact of diet was observed as significant difference was found between HP+Poly I:C+LPS and LP+Poly I:C+LPS group [*F*_(1,66)_=491, *P*≤0.001; *F*_(1,66)_=7.5, *P*≤0.001; *F*_(1,66)_=88, *P*≤0.001; *F*_(1,66)_=326, *P*≤0.001].

## DISCUSSION

Based on a novel multi-hit model, created by taking in consideration three important stressors i.e., protein malnourishment (PMN), viral and bacterial infection, it was evident that such ordeal develops Schizophrenia-like conditions during adulthood. Neuronal damage in the CA layer and extensive spine loss was found to be directly linked with downregulation of Egr 1 and Arc protein expression, further leading to a vigorous activation of microglia and astrocytes alongside their fragmentation. All these cellular degradations were accompanied with severe anti-anxiety and hyperactivity-like symptoms in the multi-hit groups, which is considered as an early hallmark of Schizophrenia.

Maternal PMN affects the diverse aspects of brain development and is associated with lasting consequences for behavioral and cognitive abilities due to altered maturation and functions of various brain areas, affecting the neurotransmitter and hormonal release (Morgane et al., 2002; Gould et al., 2018). In addition, the maternal PMN is also known to dampen the differentiation capacity of neuronal stem cells and dynamics of cell migration (Amarger et al., 2014; Gould et al., 2018). It also causes defects in neuronal circuits, augmented cell death and alteration in expression of BDNF affecting the neuronal plasticity (Morgane et al., 1993; Miranda et al., 2019). In this study as well, we have observed loss of functional spines and cellular compactness along with prominent CA layer dystrophy in LP groups. In addition, the expression of synaptic plasticity associated proteins, Egr 1 and Arc was also significantly downregulated following maternal PMN. These changes collectively indicate the severe consequence of protein deprivation on the synaptic components and neuronal circuitry formation and maturation in the hippocampus. Moreover, these changes are further deteriorated following cumulative exposure of bacterial and viral infections.

Egr 1 expression is almost absent in developing brain and increases gradually through postnatal development, reaching to adequate levels by PND 17 in rat hippocampus (McMAHON et al., 1990; Beckmann and Wilce, 1997) which corresponds the timing of synaptic formation, maximal response to NMDA and LTP inducibility respectively (Duclot and Kabbaj, 2017). Significant downregulation of Egr 1 expression in the adult hippocampus of low protein and multi-stressed groups very clearly demonstrates its consequences on synaptic components. Again, Egr 1 expression is transiently affected by a variety of stimuli through activation of MAPK/PI3K pathways and regulates the Arc expression by binding and transactivating the Arc promoter (Li et al., 2005; Duclot and Kabbaj, 2017). Thus, diminished Egr 1 expression in LP and multi-hit animals clearly depicts the lowered Arc expression in these animals.

Microglia are reported to be associated with synaptic pruning (Weinhard et al., 2018). In the present study, hype in percentage of activated microglia concomitantly expressing MHC II was seen in LP alone group which could be due to an increase in spine loss. Such microglial activation could be a reason for an overall decrease in microglial density in the LP alone group (Field et al., 2010; Han et al., 2013; Stridh et al., 2013; Chertoff, 2015; Wu et al., 2015). Earlier findings from our lab also reported that PMN affect astrogliogenesis in terms of temporal delay in GFAP emergence and precocious maturation leading to poor astrocyte number in F1 generation (Naik et al., 2017). In this study as well, a decrease in overall astrocytic population along with hype in area fraction of S100β was a prominent feature in the LP group hippocampus, indicating astrogliosis. These cellular changes due to protein malnourishment also led to behavioral deficits in terms of hyperactivity and anti-anxiety like symptoms reflecting the direct consequence of protein restriction during crucial period of life. Such behavioral deficits have been variously reported following maternal protein malnourishment (Chertoff, 2015; Naik et al., 2015; Sinha et al., 2020).

Low protein levels during development also lead to a compromised immune system (Chertoff, 2015) which cannot cope up with additional stress and this leads to a synergistic condition causing exaggerated immune response. However, the animals fed with adequate protein diet don't show such synergistic situation following additional exposure to immune stress. In our study, multi-hit group is an example of the mentioned synergistic condition but prior observing the effect of multi-hit stress, the individual effect of viral and bacterial infection on both HP and LP animals were studied to observe the effect of LP diet on single-hit exposure as well as to compare the severity of single and multi-hit stressors. The deterioration in spine density and neuronal cytoarchitecture were more in LP+Poly I:C and LP+LPS groups when compared to their HP counterparts. Studies have also reported similar neuronal changes (reduced neuronal number and dendritic complexity) on individual exposure of single type of stressor like Poly I:C and LPS (Vyas et al., 2002; Joëls et al., 2007; Richwine et al., 2008; Abazyan et al., 2010; Li et al., 2014). Again, early-life stressors can either upregulate or downregulate the Egr 1 protein expression, depending upon the mode of action (Gallo et al., 2018) and in our study, single exposure of Poly I:C and LPS to both HP and LP animals was found to trigger Egr 1 protein when compared to HP control and LP alone group respectively. Such Poly I:C mediated upregulation of Egr 1 gene in the cortex and hippocampus of rats was also reported by Baghel et al. (2018). Honkaniemi and coworkers (1994) also reported an increase in Egr 1 gene expression in hypothalamic paraventricular nucleus after capsaicin induced sensory stress response in adult Sprague Dawley rats. Such upregulation of Egr 1 protein could be due to stress induced activation in glucocorticoid receptors which further increased protein kinase activity of MAPK signaling pathway (Revest et al., 2005). LPS, on the other hand, was reported to upregulate Egr 1 expression via TNF α dependent pathway in mouse macrophage cell line RAW 264.7 involving extracellular signal regulated kinase (ERK 1/2) dependent mechanism (Shi et al., 2002). This upregulation could be a compensatory reaction of Egr 1 alone following singular exposure of either Poly I:C or LPS. These observations points to a no direct correlation between Egr 1 and Arc proteins as in the present investigation the Arc protein expression remain downregulated following single hit of either Poly I:C or LPS. As per Gröger and co-workers (2016), unpredictable stress in dual-hit rat model leads to downregulation of Egr 1 expression and upregulation of Arc expression, 2 h. post stress exposure. Also, the early wave of Arc expression in hippocampus following electroconvulsive shock was found to be unaffected in Egr 1 gene mutant mice, further suggesting independent role of Egr 1 gene (Penke et al., 2011). Furthermore, LPS exposure is also reported to lower the expression of Arc in cortex of treated rats, further suggesting infection related downregulation of Arc protein (Bonow et al., 2009). Other nutritional abnormalities like high fat diet have also been reported to decrease Arc level in cerebral cortex by reducing tyrosine phosphorylation of NMDAR2A and NMDAR1 (Mateos et al., 2009). Thus, the compensatory upregulation in Egr 1 expression following Poly I:C or LPS is not able to influence the impaired Arc expression in the stressed groups.

The non-neuronal cells of the hippocampus also responded vigorously to the exposure of early-life stressors like Poly I:C and LPS. HP animals following single hit of either Poly I:C or LPS showed an increase in both activated microglia and astrocytes. Many of the activated microglia and astrocytes also labeled strongly for MHC II and S100β protein respectively, suggesting their transformation into activated cells (Patel et al., 1999; Kaur et al., 2017). Whereas, in LP+Poly I:C and LP+LPS groups, both activation and degeneration of microglia and astrocytes were observed which caused a decrease in total cell population when compared to the HP+Poly I:C and HP+LPS groups. Thus, the contrasting difference in the population of MHC II and S100β expressing cells in the LP and HP groups were directly correlated with the decrease in glial density following maternal protein deprivation as also reported by Spencer et al. (2017). Lastly, the behavioral deficits in Poly I:C and LPS exposed LP animals were also more than their HP counterparts and HP and LP alone groups. Similar to this observation, studies carried through exposure to maternal protein malnutrition or neonatal infections (Poly I:C and LPS) have also shown deterioration of behavior as detected by EPM and OFT experiments (Meyer, 2014; Naik et al., 2015; Singh et al., 2017; Baghel et al., 2018; Sinha et al., 2020).

Finally, synergistic impact of low protein, Poly I:C and LPS (multi-hit) caused more damage to the nervous system when compared to the remaining groups. Severe spine loss was also seen in the multi-hit group along with formation of dendritic varicosities which is responsible for spine engulfment and could be the reason for minimal spines in the multi-hit group (Šišková and Tremblay, 2013; Kanamori et al., 2015). Spine loss on multi-hit exposure along with vigorous increase in pyknotic neurons and apoptotic bodies could be associated with severe downregulation of Egr 1 and Arc protein. Although, no multi-hit related study is reported so far but these observation tally with results reported on single exposure of Poly I:C and LPS (Kondo et al., 2011; Bai et al., 2015; Zhang et al., 2015; Li et al., 2015; Burrows et al., 2016; Qiao et al., 2016). In our study, the LP+Poly I:C+LPS animals showed severe downregulation of Egr 1 protein and similar to this, chronic stress induced downregulation of Egr 1 was reported by Matsumoto et al. (2012), Okada et al., (2014), Ieraci et al. (2016) and Zhang et al., (2018). They further suggested that Egr 1 gene downregulation can be related to various neurological disorders like Schizophrenia. To our understanding, stress-related downregulation observed in the present study could be because of habituation caused by repeated stress exposure or due to incapability of LP animals to respond to repeated stress. These observations are in line with the reports of Girotti and co-workers (2006), suggesting reduced expression of immediate early gene on habituation with repeated restrain stress exposure.

Again, the percentage of activated microglia was maximum in LP multi-hit group suggesting hyper-activation of microglia which could also be the reason for stress dependent microglial fragmentation prominently seen in LP+Poly I:C+LPS group. Also, the HP and LP animals did not synchronize on multi-hit of Poly I:C and LPS. While both astrocytic number and GFAP expression increased in HP+Poly I:C+LPS, indicating astrogliosis, the same was decreased in LP+Poly I:C+LPS animals, may be due to widespread astrocytic degeneration. Following immune challenge with either Poly I:C or LPS to LP animals, initially there was an astrocytic response in terms of hyperplasia, but cumulative exposure led to their degeneration, indicating the inability of LP animals to cope up with multiple stress exposure. Such astrocytic degeneration has also been reported in conditions of chronic immobilization stress and LPS exposure (Sharma et al., 2016; Naskar and Chattarji, 2019). Astrogliosis was further confirmed by S100β labeling and an upregulation of S100β protein was seen in multi-hit groups (Sorci et al., 2010; Serrano et al., 2017).

All these cellular changes and behavioral abnormalities which were severe in multi-hit group are also seen in Schizophrenic conditions (Glausier and Lewis, 2013; Comer et al., 2020). And in the light of these findings, the multi-hit rats clearly indicate a far-reaching implication for developing Schizophrenia-like phenotype.

### Conclusion

This study hereby concludes that synergistic exposure of multiple perinatal stressors causes more damage than any single stressor. Such cumulative and chronic stress accelerates neuronal degeneration, synaptic loss alongside downregulation of synaptic plasticity associated genes, glial alteration and behavioral deficits, mimicking the Schizophrenia like pathology at adulthood. Present study thus suggests that exposure to multiple early-life stressors increases the risk for developing Schizophrenia at adulthood (Fig. S1).

## MATERIALS AND METHODS

### Animal husbandry and perinatal stress exposure

**Fig. 8.**
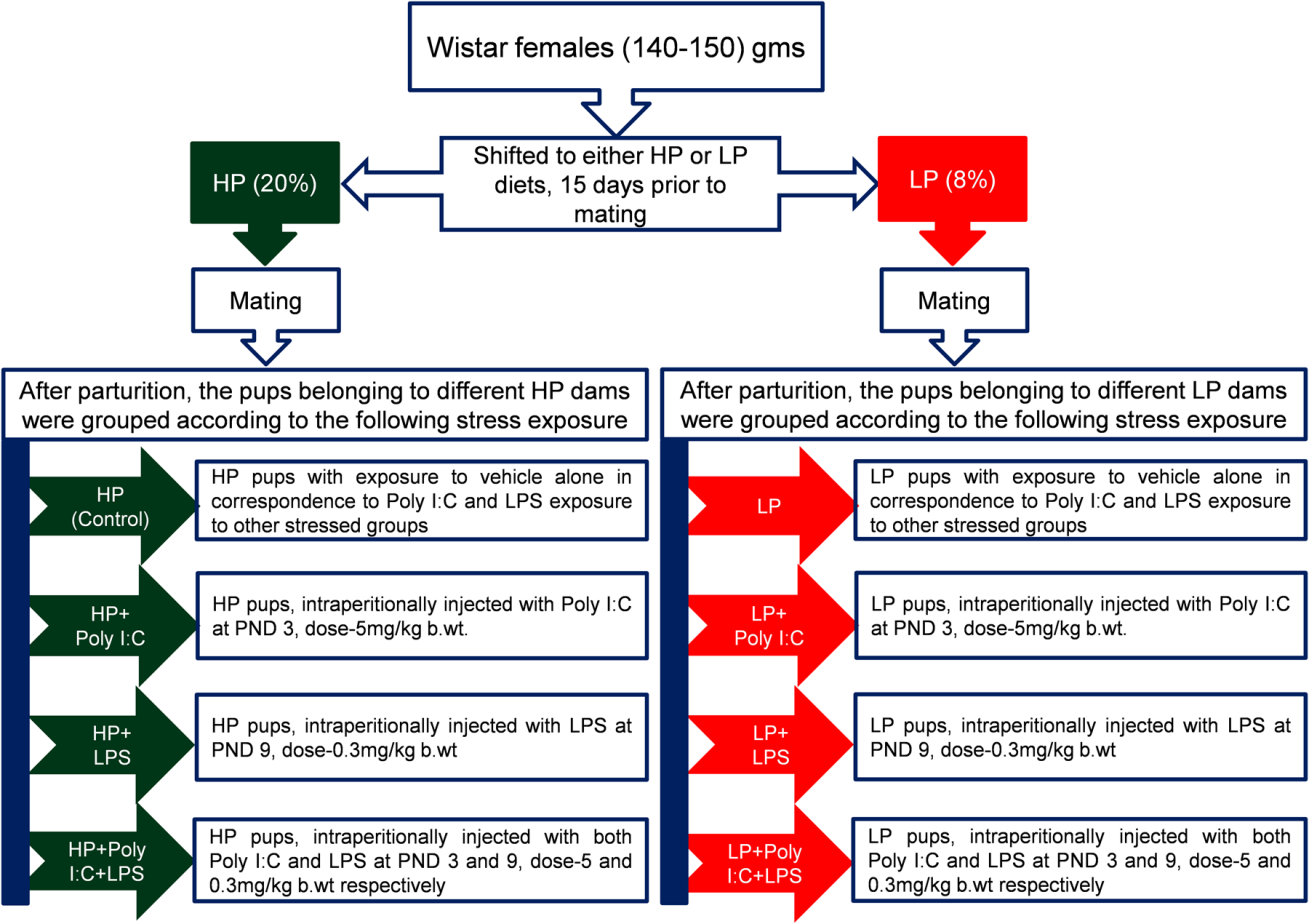
Work plan represented in a flowchart.

Wistar rats maintained in controlled physical environment (temperature=25±1°C, humidity=65±2%, light and dark cycle=12 h) were used to create the stressed models. Prior to shifting to the experimental diets, all F_0_ (*n*=32) females were given *ad libitum* access to reverse osmosis (RO) water and standard rat pellet feed. Equal numbers of 3-month-old female rats (140-150 g) were shifted to HP control (*n*=16, high protein, 20%) and LP (*n*=16, low protein, 8%) diets, 15 days prior to mating and maintained on the same diets throughout gestation as well as lactation. The day of parturition was noted as postnatal day (PND) 0. The F_1_pups born to both HP control and LP females were used to create the following groups (Fig. 8). Litter size was adjusted to eight pups per dam to avoid variability due to varying litter size.

### HP (control) and LP alone groups

For control (HP) group, pups (*n*=32) obtained from four different HP dams without any stress were used. While LP alone group contained pups (*n*=32) born to four different LP females without any further exposure of stressors.

### Poly I:C (viral mimetic) and LPS (Lipopolysaccharide, bacterial endotoxin) preparation

Poly I:C stock solution was prepared by dissolving 5 mg of Poly I:C (P1630, Sigma-Aldrich; St. Louis, MO, USA) in pre-heat (60°C) TBE buffer (Tris-Borate-EDTA, pH-8). The solution was vortex mixed, centrifuged and stored at 4°C for further use. For LPS stock solution, 0.3 mg LPS (L2630, Sigma-Aldrich, *E. coli*, serotype 0111:B4) was dissolved in 1 ml of PBS (Phosphate buffer saline, pH-7.2) and stored at 4°C for use.

### HP+PolyI:C and LP+Poly I:C groups

Viral infected HP and LP models (HP+Poly I:C; LP+Poly I:C) included intraperitoneally Poly I:C (viral mimetic) injected HP and LP pups (*n*=32 each) from four different HP and LP dams at PND 3 at a dosage of 5 mg/kg body wt.

### HP+LPS and LP+LPS groups

To create bacterial infected HP and LP models (HP+LPS; LP+LPS), LPS (bacterial mimetic) was injected intraperitoneally to pups obtained from four different HP and LP diet fed females (*n*=32 each) at PND 9 at a dosage of 0.3 mg/kg body wt.

### HP+Poly I:C+LPS and LP+Poly I:C+LPS (multi-hit) groups

For multi-hit model i.e., viral and bacterial combined HP and LP groups (HP+Poly I:C+LPS; LP+Poly I:C+LPS), pups from four different females fed with HP and LP diets (*n*=32 each) were injected intraperitoneally with Poly I:C at PND 3, followed by LPS at PND 9 at the doses mentioned vide supra.

Following intraperitoneal injections, the pups were immediately returned to their respective dams and after weaning at PND 21, were maintained on their respective diets till used at PND 180, according to the experimental plan. All injecting procedures were done under aseptic conditions using Stoelting Nanoinjector and Hamilton micro syringe at a controlled rate to maintain accuracy and complete absorption. Controls were injected with vehicle alone with respect to Poly I:C and LPS injections. Experimental plan was designed with prior permission from Institutional Animal Ethics Committee of Jiwaji University, Gwalior (M.P), India. Registration number: 1854/GO/Re/S/16/CPCSEA.

### Tissue harvesting

Brain tissues for histology and immunohistochemistry were harvested using perfusion technique in which F_1_ animals (*n*=6 animals, from four different dams per group) from each group at PND 180 were anesthetized with diethyl ether and transcardially perfused using pre-chilled PBS (phosphate buffer saline, 0.01 M, pH-7.4) followed by fixative i.e., 2% paraformaldehyde in 0.01 M PB (Phosphate buffer) to obtain cryosections and 10% buffered formalin for paraffin sections. The medial temporal lobe region was dissected out and immersed overnight in the same fixative used for perfusion. For cryosectioning, the tissues were maintained at 4°C, subsequently cryoprotected with sucrose gradients (10%, 20% and 30% sucrose in PB) and then sectioned (coronal, 14 µm thick) using a cryotome machine (Leica CM1900, Germany), followed by storage at −20°C for immunolabelling procedures (Table S2).

For paraffin sectioning, after post-fixation at room temperature, the tissues were washed with dH_2_O, dehydrated using alcohol series (30%, 50%, 70%, 90%, 100%), cleared with toluene and then immersed in molten paraplast (Sigma-Aldrich, m.p. 57°-58°C) at 57°C in a pre-heated oven, followed by block mapping. After solidification, the tissue blocks were cut using a microtome machine (Leica RM 2135, 10 µm thick coronal sections) and the slides containing the microtome cut hippocampal sections were air dried for 72 h in a dust-free room and stored in a cool place until used for cresyl violet (CV) staining.

### Golgi impregnation method for morphological analysis of neurons

The animals from each group (*n*=6 animals, from four different dams/group) were decapitated, their forebrains were dissected out and immediately immersed fixed in Golgi fixative (solution of sodium dichromate, chloral hydrate, formaldehyde, glutaraldehyde and DMSO in dH_2_O) for 72 h, followed by treatment with 0.75% silver nitrate solution for 48 h. After impregnation with silver nitrate, the coronal sections were cut (100 µm thick) using a vibratome machine (Leica, VT 1000s). The cut sections were collected in cavity blocks, dehydrated with alcohol series, cleared with xylene and mounted with DPX. The slides were air dried and stored for analysis.

### Nissl staining using CV stain

For histological study of neurons, paraffin sections (*n*=6, from six different animals/group) were deparaffinized in xylene, hydrated in alcohol series and stained in 0.1% CV solution, prepared by dissolving cresyl violet (Sigma-Aldrich certified stain, C-5042) in acetate buffer (pH-3.5). The sections were quickly dehydrated in n-butyl alcohol, cleared in xylene and mounted with DPX. The slides were stored at room temperature for analysis.

### Anti-Egr 1 and Anti-Arc immunofluoresence labeling

Separate batches of slides were used for Egr 1 and Arc labeling and the below mentioned protocol was followed for both antibodies. Slides containing cryocut coronal sections of different rat brains through hippocampus were randomly selected (*n*=6 slides from six different animals/group) for each parameter and air dried for 45 min. The slides were rinsed with PBS to remove cryomount and then treated with 1% Triton X-100 in PBS for membrane permeabilization for 20 min. Subsequently the slides were washed with PBST (PBS+0.1% Tween 20) and incubated with 10% normal goat serum (NGS; Vector) in PBS for 2 h at room temperature for non-specific protein blocking. This was followed by incubation with primary antibodies, anti-Egr 1 (1:100 in 5% BSA in PBST, rabbit polyclonal; Santacruz, SC189) and anti-Arc (1:100 in 5% BSA in PBST, mouse monoclonal; Santacruz, C-7:SC17839) for 48 h at 4°C in a humid chamber. After primary antibody incubation, the sections were brought to room temperature, washed in PBST and incubated with fluorochrome labeled secondary antibodies at a concentration of 1:200 in 5% BSA in PBST (anti-rabbit Alexa Fluor 488for Egr 1; anti-mouseAlexa Fluor 594 for Arc) for 2 h at room temperature in a dark and humid chamber. After secondary antibody incubation, the sections were washed in PBS (five changes of 10 min each) for removal of unbound secondary antibodies and mounted with antifade Vectashield Hard Set mounting medium with DAPI (Vector laboratories, CA, USA). The slides were finally visualized with fluorescence microscope using specific filters.

### Immunohistochemical labeling of GFAP, S100β, Iba1 and MHC II (OX 6) proteins using streptavidin biotin HRP method

Following protocol was followed for immunolabeling of Glial fibrillary acidic protein (GFAP), S100β, ionized calcium binding adapter molecule 1 (Iba1) and MHC II (OX6) proteins using specific antibodies. Slides containing (*n*=6 slides from six different animals/group) cryocut sections containing the hippocampus region from each group were selected and air dried for 45 min. The sections were washed with PBS for GFAP, S100β and Iba1 immunolabeling and TBS (Tris buffered saline, pH 7.4-7.6) for MHC II immunolabeling followed by incubation with Triton X-100 (Sigma-Aldrich) for permeabilization. The sections were then washed with PBS (GFAP, S100β and Iba1) or TBS (MHC II), followed by blocking with 1% H_2_O_2_in the respective buffers for 20 min. Subsequently the sections were incubated with 1% serum (Normal Serum, Vector kit PK6101 for GFAP and Iba1; Vector kit PK6200 for S100β and MHC II) for 90 min, followed by overnight incubation with primary antibody i.e. anti-GFAP (1:1000, rabbit polyclonal, Z0334 Dako), anti-S100β (1:500, mouse monoclonal, S2532, Sigma), anti-Iba1 (1:800, rabbit polyclonal, 019-19741, Wako) and anti-OX-6, (MHC II; 1:150, mouse monoclonal, MCA46G, Serotec) at 4°C. Next day, the sections were brought to room temperature and rinsed with PBS (GFAP, S100β and Iba1) or TBS (MHC II) for removal of unbound primary antibodies. The sections were then incubated with secondary antibodies for 2 h (1:100, Vector kit PK6101 for GFAP and Iba1; 1:100, Vector kit PK6200 for S100β and MHC II) followed by PBS (GFAP, S100β and Iba1) or TBS (MHC II) washing and then incubated with streptavidin biotin HRP complex for 2 h (1:200, Vector kit PK6101 for GFAP and Iba1; 1:200, Vector kit PK6200 for S100β and MHC II). The sections were then washed with PBS/TBS followed by treatment with DAB solution (25 mg DAB+60 µl H_2_O_2_ in 100 ml of PBS/TBS) for 20 min for visualization. Additionally, for better visualization, nickel was added with DAB solution during MHC II immunolabelling. The reaction was finally terminated under running tap water and sections were air dried, dehydrated, cleared with xylene and mounted with DPX. The prepared slides were air dried and stored for analysis (Sharma et al., 2016).

### Image analysis

Bright field images for histological and immunohistochemical studies were grabbed using Leica DFC I310 FX digital camera connected to Leica DM 6000 microscope, incorporated with Leica Application Suite (LAS V4.2) software. For fluorescence imaging of Egr 1 and Arc labeling, Leica DM 6000 microscope connected to Leica DFC 310 IFX digital camera and operated with LAS/AF, (Advanced Fluorescence) software was used. The images were grabbed using I3 and N2.1 filter for Alexa Fluor 490 and 596 respectively.

For image quantification, images with constant frame area (21670.9 µm^2^) and magnification (20X) were grabbed from different areas of hippocampus (*n*=108, from six different slides belonging to six different animals per group). The area fraction (% positively labeled areas) and cell count (cells/mm^2^) representing protein density and cell number in each frame of interest were measured using image quantification and interactive module of Leica Qwin software (V3.1). Fluorescence intensity was measured from grabbed images using LAS/AF software. Data for different regions of hippocampus (CA1, CA3 and DG) were compiled and represented as histograms.

Golgi images on the other hand were grabbed using oil microscopy (Leica DFC 420IC digital camera connected to Leica Laborlux microscope) at 100X magnification. Spine density in the distal 100 µm length of secondary basal dendrites of pyramidal neurons was measured using Leica Qwin software, by counting the number of spines in the defined dendrite (*n*=108, six neurons per slide were analyzed for each group).

### Behavioral tests

#### Elevated plus maze (EPM)

Equal number of animals (*n*=12, from different dams per group) from each group were acclimatized to the behavioral room, 2 h prior to testing and then analyzed for behavioral abilities through EPM. EPM is a plus shaped elevated (75 cm) maze consisting of two closed and two open arms. The test animals were individually placed in the center area of EPM and allowed to explore the maze area for 120 s. Three trials were conducted for each animal with 2 h gap between each trial. Data were recorded as the time spent and the number of entries in both open and closed arms, using vertical camera attached with computer loaded with Any Maze software (v4.82). The percent time spent and number of entries (%) in open arms was calculated by the following formula: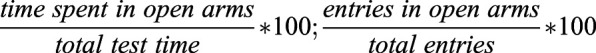 respectively.

#### Open field test (OFT)

The apparatus used is a transparent acrylic box (42 cm×43 cm×22 cm) consisting of two arrays of infrared beams perpendicular to each other (30 infrared photocells, 15 per row) to detect vertical and horizontal locomotor activity of rats. The apparatus was connected to a computer equipped with Optovarimax Autotrack activity monitoring software (Columbus Instruments, OH, USA, v4.41). Each animal (*n*=12, from four different dams per group) was pre-exposed to the open arena for 5 min to rule out anxiety created on exposure to any novel environment. Locomotor behavior of each animal was recorded for 20 min using the mentioned software and the following variables were recorded for interpretation of behavior in OFT: distance travelled (cm), resting time (s), ambulatory time (s) and stereotypic time (s).

### Sample size determination

Both male and female rats were randomly selected at PND 180 for all the investigation performed.
Equal numbers of male and female animals (*n*=12 per group) were used for behavioral experiments as no sex specific difference was obtained. For Golgi method, immunofluoresence and immunohistological studies, *n*=6 animals from different dams per group was used.
For quantification analysis of immunofluorescence (*n*=36) and immunohistochemical data (*n*=108) images were grabbed from six different slides obtained from six animals per group. The mean data were then represented graphically.
To ensure adequate power, sample size was determined based upon *P*-value, power, effect and alternative hypothesis (Suresh and Chandrashekara, 2012).
Animals with cervical dislocation and sickness like behavior were excluded. All healthy animals were included for all the experiments, keeping the *n* number constant.

### Statistical analysis

All data were statistically analyzed using one-way (for groupwise interaction) and two-way (for interaction between two independent variables i.e. diets and treatment) ANOVA, followed by Holm-Sidak *post hoc* test. Sigma Plot version 12.0 was used for analysis and significance level was preset at *P*≤0.05.

## Supplementary Material

Supplementary information
