# Supplementary material for: Perinatal exposure to synergistic multiple stressors leads to cellular and behavioral deficits mimicking Schizophrenia-like pathology
Source: Biol Open. 2022 Mar 9;11(3):bio058870. doi: 10.1242/bio.058870 (PMC8918990; doi:10.1242/bio.058870)
Supplement: Supplementary information [file biolopen-11-058870-s1.pdf]

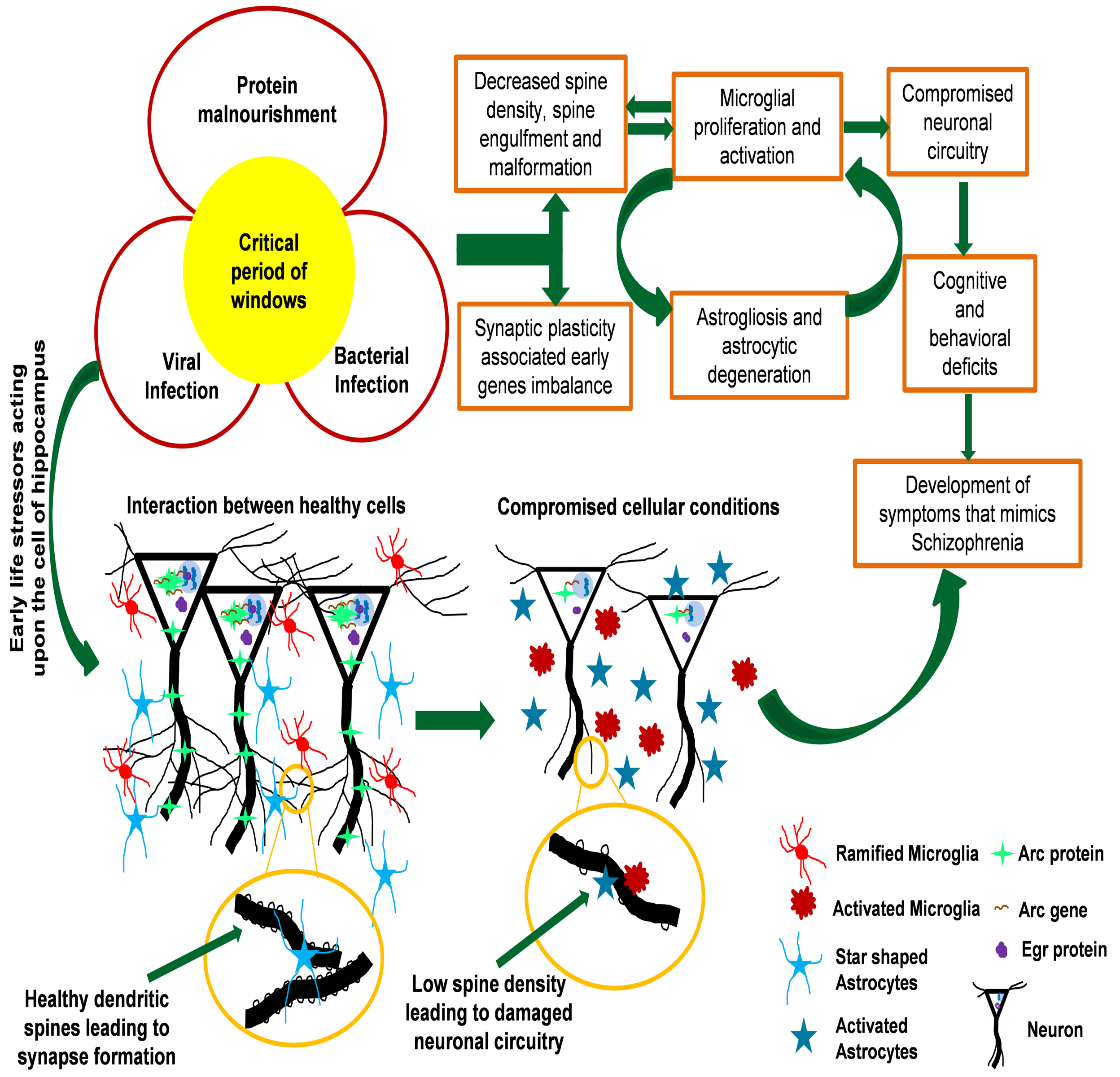

Fig. S1.

Biology Open • Supplementary information

Table S1. Antibody details

Sl no.	Name of the Antibody	Catalogue number/clone number	Batch number	Citation/antibody validation profile	Dilution
1.	Egr 1	SANTA CRUZ BIOTECHNOLOGY (C-19): sc-189/rabbit polyclonal	K2315	Song et al., 2015; Sun et al., 2019	1:100
2.	Arc	SANTA CRUZ BIOTECHNOLOGY C-7:sc-17839/mouse monoclonal	E0914	Scarlata et al., 2019; Bye and McDonald, 2019	1:100
3.	GFAP	Dako Z0334/rabbit polyclonal	00031729	Kriszta et al., 2019	1:1000
4.	S100b	Sigma S2532/mouse monoclonal	115K4846	Duda et al., 2017	1:500
5.	Iba 1	Wako 019-19741/rabbit polyclonal	PTR2404	Rodriguez-Callejas et al., 2016	1:800
6.	OX 6	Serotec MCA46G/mouse monoclonal	0109	Zilka et al., 2009	1:150

Reference

Bye, C.M. and McDonald, R.J. (2019). A specific role of hippocampal NMDA receptors and arc protein in rapid encoding of novel environmental representations and a more general long-term consolidation function. Front. Behav. Neurosci. **13**, 8.

Duda, I., Krzych, Ł., Jędrzejowska-Szypułka, H. and Lewin-Kowalik, J. (2017). Serum levels of S100B protein and neuron-specific enolase are associated with mortality in critically ill patient. Acta Biochim. Pol. **64**, 647-652.

Kriszta, G., Nemes, B., Sándor, Z., Ács, P., Komoly, S., Berente, Z., Bölcskei, K. and Pintér, E. (2020). Investigation of cuprizone-induced demyelination in mGFAP-driven conditional transient receptor potential ankyrin 1 (TRPA1) receptor knockout mice. Cells **9**, 81.

Rodriguez-Callejas, J.D., Fuchs, E. and Perez-Cruz, C. (2016). Evidence of tau hyperphosphorylation and dystrophic microglia in the common marmoset. Front. Aging Neurosci. **8**, 315.

Biology Open • Supplementary information

Scarlata, M.J., Lee, S.H., Lee, D., Kandigian, S.E., Hiller, A.J., Dishart, J.G., Mintz, G.E., Wang, Z., Coste, G.I., Mousley, A.L. and Soler, I. (2019). Chemogenetic stimulation of the infralimbic cortex reverses alcohol-induced fear memory overgeneralization. Sci. Rep. **9**, 1-15.

Song, Y., Cheng, X., Yang, X., Zhao, R., Wang, P., Han, Y., Luo, Z., Cao, Y., Zhu, C., Xiong, Y. and Liu, Y. (2015). Early growth response-1 facilitates enterovirus 71 replication by direct binding to the viral genome RNA. Int. J. Biochem. Cell Biol. **62**, 36-46.

Sun, Z., Xu, X., He, J., Murray, A., Sun, M.A., Wei, X., Wang, X., McCoig, E., Xie, E., Jiang, X. and Li, L. (2019). EGR1 recruits TET1 to shape the brain methylome during development and upon neuronal activity. Nat. Commun. **10**, 1-12.

Zilka, N., Stozicka, Z., Kovac, A., Pilipcinec, E., Bugos, O. and Novak, M. (2009). Human misfolded truncated tau protein promotes activation of microglia and leukocyte infiltration in the transgenic rat model of tauopathy. J Neuroimmunol. **209**, 16-25.
